# GlomNet: glomeruli segmentation network for WSI with diverse staining protocols for kidney disease diagnosis

**DOI:** 10.3389/fmed.2026.1785037

**Published:** 2026-04-13

**Authors:** Waseem Hameed, Guangchao Yang, Muhammad Wajeeh Us Sima, Muhammad Arshad, Salem Alkhalaf, Fahad Alturise

**Affiliations:** 1 Department of Computer Science and Technology, Chongqing University, Chongqing, China; 2 Department of Computer Engineering, College of Computer, Qassim University, Buraydah, Saudi Arabia; 3 Department of Cybersecurity, College of Computer, Qassim University, Buraydah, Saudi Arabia

**Keywords:** artificial intelligence, glomeruli segmentation, renal pathology, Transformers, whole-slide images

## Abstract

Accurate segmentation of glomeruli in kidney histopathology images is vital for diagnosing renal diseases but remains challenging due to several limitations in current CNN-based methods. First, multiscale learning struggles to preserve the critical features of small glomeruli, as uniform processing dilutes their discriminative information and hinders effective separation from surrounding tissue. Second, limited interaction between consecutive layers leads to incomplete feature fusion, weakening the representation of complex glomeruli structures. Finally, traditional upsampling techniques introduce artifacts like checkerboards and blurred edges, further compromising segmentation accuracy. To address these challenges, we propose GlomNet, a simple yet effective glomeruli segmentation network designed for whole-slide images (WSIs) with diverse staining protocols, enabling accurate segmentation for Kidney disease diagnosis. GlomNet integrates three key components: Local-Global Cris-Cross Former (LGC^2^-Former) efficiently captures global contextual information and fine-grained local details by processing the input feature map through parallel global and local branches. It then aggregates these contextual cues along both horizontal and vertical directions, enhancing spatial awareness and improving segmentation accuracy of complex glomeruli structures. Hierarchical Multi-head Feature Aggregation (HMFA) promotes richer feature extraction and effective multi-scale fusion by enabling mutual guidance between consecutive layers, addressing the underutilization of complementary feature information. Feature-Refined Upsampling (FRU) resolves issues related to checkerboard artifacts and blurry edges in traditional upsampling methods, while improving resolution and maintaining feature integrity during the decoding phase for more accurate segmentation. Experimental results demonstrate that GlomNet achieves state-of-the-art performance across multiple benchmarks, including NEPTUNE, HuBMAP-1, and HuBMAP-2, showcasing its robustness across diverse histological conditions, with significant potential for real-world applications in the diagnosis of kidney disease and medical image analysis.

## 1 Introduction

The glomerulus is a vital functional unit of the human kidney, playing a central role in blood filtration (1). Damage to glomeruli caused by primary or systemic diseases can lead to renal failure, posing a serious threat to patient health and creating a substantial burden on healthcare systems. Accurate assessment of glomerular structures is therefore essential for early diagnosis, effective treatment planning, and monitoring disease progression. Manual segmentation of glomeruli, however, is highly labor intensive, time consuming, and prone to variability, making it impractical for large scale clinical applications. Traditional approaches have relied on handcrafted features such as spectral, spatial, and textural descriptors combined with classical machine learning models, including Support Vector Machines and Random Forests (2). While these methods have provided initial solutions, their dependency on manually designed features often results in fragmented or inaccurate segmentations. This highlights the urgent need for robust, deep learning-based methods capable of capturing the intricate structures of glomeruli and surrounding microvascular networks with high precision and reproducibility.

With the widespread adoption of Convolutional Neural Networks (CNNs) in computer vision, significant advancements have been achieved in semantic segmentation. Fully Convolutional Networks (FCNs) (3) pioneered end-to-end dense pixel prediction by removing fully connected layers, enabling precise segmentation across entire images. U-Net (4) introduced skip connections in an encoder decoder framework to preserve spatial details, while U-Net++ (5) incorporated dense skip connections to bridge the semantic gap between encoder and decoder feature maps. Architectures such as PSP-Net (6), using the Pyramid Pooling Module (PPM), and DeepLabV3+ (7), employing Atrous Spatial Pyramid Pooling (ASPP), have demonstrated strong multiscale segmentation capabilities, effectively capturing both local and global contextual information. Despite these advances, applying CNN-based methods to kidney histopathology images still faces significant challenges. Multiscale feature extraction in multiscale learning often fails to preserve the sparse yet critical features of glomeruli, with limited interaction between consecutive layers (8). Furthermore, traditional upsampling methods can lead to checkerboard artifacts or blurred edges, ultimately diminishing segmentation accuracy. Addressing these challenges is essential for developing automated systems capable of precise and reliable glomerular segmentation in renal tissue images. Recent pure CNN-based approaches for glomeruli segmentation have focused on optimizing feature pyramids and standard U-Net backbones to handle morphological variability without the computational weight of Transformers. Juang et al. (9) proposed a Flattened Xception network integrated with a Feature Pyramid Network (FX-FPN), utilizing generative morphology augmentation (CycleGAN) to significantly improve the detection and classification of varying glomerular shapes in multi-stained images. Similarly, Addressing the specific challenge of sclerotic tissue, Souza et al. (10) extensively evaluated U-Net and U-Net3+ architectures, demonstrating that while these pure CNN models achieve high accuracy (Dice > 0.94) on healthy glomeruli, they struggle significantly with global glomerulosclerosis due to the loss of boundary cues, highlighting the limits of purely convolutional receptive fields.

To address the limitations of CNN-based models in capturing long-range dependencies, recent research between 2023 and 2025 has increasingly shifted toward hybrid architectures that synergize the local feature extraction of CNNs with the global contextual understanding of Transformers. While CNNs excel at delineating local boundaries critical for the precise segmentation of glomeruli they often fail to model the broader tissue context required to distinguish complex histological structures. To bridge this gap, He et al. (11) introduced H2Former, a hierarchical hybrid transformer that integrates multi-scale channel attention with CNNs to model global dependencies without losing local detail, a concept further refined by HyFormer (12), which employs a hybrid strategy to capture target structures that span widely across images. Building on the success of the Swin Transformer in handling multi-scale variations, recent works like FE-SwinUper(13) and MS-Former (14) have demonstrated that integrating Swin blocks with U-Net like decoders significantly enhances feature representation across different resolutions, a critical factor for segmenting variable-sized glomeruli. Specifically in the domain of kidney pathology, PST-UNet (15) proposed a fusion of Swin Transformers and U-Net to better preserve spatial features of renal lesions, while Mask2Former with a Swin backbone (16) was shown to outperform traditional UNet-ResNet models in segmenting kidney compartments by effectively handling inflammation and fibrosis. Further innovations in attention mechanisms include DA-TransUNet (17), which integrates spatial and channel dual attention to refine boundary detection, and MMAformer (18), which utilizes a multi-stage encoder to fuse complementary information at varying scales. Similarly, FGSSM (Fine-grained Scaling Segmentation Model) (19) addressed the challenge of diverse tissue patterns in histopathology by enhancing U-Net with dual classifier pairs. These hybrid approaches, including the L-MLSTM (20) and RCSHT (Robust Cross-Scale Hybrid Transformer) (21), collectively illustrate a paradigm shift toward architectures that leverage cross-scale interactions and self-attention to resolve the intricate morphologies typical of renal histology.

Despite significant advances semantic segmentation, challenges remain in applying these methods to kidney histopathology images. First, multiscale learning approaches struggle to retain the sparse yet critical information of tiny glomeruli. The uniform processing of irrelevant features dilutes their discriminative characteristics, hindering effective separation from surrounding tissue across multiple scales. Second, limited interaction between consecutive layers leads to underutilization of complementary feature information. As adjacent layers independently extract features, this results in incomplete fusion and weaker representations for complex renal structures, including glomeruli and microvascular networks. Finally, conventional upsampling techniques, such as deconvolution or bilinear interpolation, can introduce checkerboard artifacts and blurred edges, further limiting segmentation accuracy.

To overcome these limitations, we propose GlomNet, a simple yet effective glomeruli segmentation network designed to handle diverse staining protocols in whole-slide images (WSIs) for Kidney disease diagnosis. GlomNet consists of three key components that collectively address these challenges. The Local-Global Cris-Cross Former (LGC^2^-Former) splits the input feature map into two parallel streams. The global context branch captures long-range dependencies by analyzing row-wise feature patterns and content-based similarities across distant regions, while the local detail branch preserves fine-grained glomerular features by modeling the residual between local features and the global context. These streams are fused, and attention is computed along both horizontal and vertical directions, enhancing spatial awareness and enabling precise segmentation of complex renal structures across diverse staining modalities. The Hierarchical Multi-head Feature Aggregation (HMFA) module addresses the underutilization of complementary feature information between consecutive layers. By enabling mutual guidance between adjacent layers, HMFA promotes richer feature extraction and effective fusion of multi-scale information, improving the representation of complex renal structures, including tiny glomeruli and microvascular networks. Finally, the Feature-Refined Upsampling (FRU) Module improves the resolution of feature maps in the decoder while fully integrating multi-scale information. Unlike conventional upsampling methods, FRU mitigates checkerboard artifacts and blurry edges, enhancing overall network performance. Together, these components enable GlomNet to robustly capture fine-grained details and global contextual relationships, addressing the key limitations of prior CNN-based methods and delivering accurate, efficient segmentation of glomeruli and associated renal structures in WSIs.

The contributions of this work are summarized as follows.

1. A simple yet effective glomeruli segmentation network, GlomNet, is designed for WSIs with diverse staining protocols, enabling accurate segmentation across varying histological conditions for Kidney Disease Diagnosis.
2. LGC^2^-Former efficiently captures global contextual information and fine-grained local details by processing the input feature map through parallel global and local branches. Attention is computed on these fused contexts, aggregating contextual cues along both horizontal and vertical directions, which enhances spatial awareness and improves segmentation accuracy of complex glomeruli structures.
3. HMFA promotes richer feature extraction and effective multi-scale fusion by enabling mutual guidance between consecutive layers, addressing the underutilization of complementary feature information.
4. FRU resolves issues related to checkerboard artifacts and blurry edges in traditional upsampling methods, while improving resolution and maintaining feature integrity during the decoding phase for more accurate segmentation.

## 2 Related work

The first notable approach is the Fully Convolutional Network (FCN) (3). Although FCNs enable end-to-end semantic segmentation, they suffer from significant spatial information loss during downsampling. As a result, recovering fine-grained details during upsampling becomes challenging, even when transposed convolutions are employed, ultimately leading to coarse segmentation output. To address gradient degradation issues, Ronneberger et al. (4) introduced the U-Net architecture, which incorporates skip connections between the encoder and decoder to facilitate improved gradient flow and better preservation of spatial details.Despite this advancement, the use of fixed convolutional kernels in U-Net results in limited receptive fields, making it difficult to effectively capture multiscale contextual information. To overcome this limitation, Zhao et al. (22) proposed the Pyramid Pooling Module (PPM), which aggregates contextual information from multiple pooling scales to generate multiscale feature representations. However, the use of pooling operations may lead to the loss of fine pixel-level details, as neighboring pixels can share identical contextual representations. Building on this concept, Chen et al. (7) proposed DeepLabv3+, which introduces an Atrous Spatial Pyramid Pooling (ASPP) module. ASPP employs parallel atrous convolutions with varying dilation rates to capture multiscale features more effectively. Nevertheless, this approach faces limitations when handling extreme scale variations. Moreover, excessively high dilation rates can introduce gridding artifacts and sparse sampling effects, which may result in partial loss of spatial information.

### 2.1 Encoder

AlexNet (23) was one of the first deep convolutional neural networks (CNNs) to demonstrate the effectiveness of stacking convolutional filters on image data to learn hierarchical feature representations, achieving significantly better performance than traditional computer vision methods. Building upon this idea, VGGNet (24) introduced deeper architectures using smaller convolutional kernels, which not only increased network depth but also effectively expanded the receptive field at higher layers. This design enabled VGGNet to capture finer multiscale information compared to AlexNet. Nevertheless, both AlexNet and VGGNet employ relatively static receptive fields at each layer, limiting their ability to effectively model features across varying spatial scales. To address this limitation, Szegedy et al. (25) proposed InceptionNet-v4, which utilizes parallel convolutional filters of different sizes within the same layer to enlarge the effective receptive field. Furthermore, Inception-ResNet-v2 integrates inception modules with residual connections, facilitating efficient information flow and improved learning of deep hierarchical representations. However, small-scale features remain highly sensitive to subsampling and may be lost when the size of critical structures is relatively small. To preserve such information, backbone networks are often designed to encode fine-grained details in the early layers and propagate these representations to deeper stages. This baseline encoding is essential for aggregating both local and global contextual information.

Local feature interactions enable networks to learn structure-specific patterns within small regions, reducing vulnerability to subsampling, while global interactions facilitate information exchange across distant regions of the image. Recent advances in Transformer-based architectures for computer vision highlight the importance of balancing local and global feature modeling, particularly for tasks such as Tiny Object Detection (TOD), where both fine details and broader contextual awareness are critical. The Vision Transformer (ViT) (26) employs multi-head self-attention (MSA) to model global dependencies; however, its quadratic computational complexity has motivated the development of more efficient alternatives. The Swin Transformer (27) addresses this issue by restricting self-attention to local windows, reducing computational complexity from $O\left(N^{2}\right)$ to O(N), while shifted window mechanisms enable inter-window communication. Extending this idea, the Shuffle Transformer (28) enhances interactions between neighboring windows through spatial shuffling, and HaloNets (29) introduce a local pixel “halo” to jointly model fine-grained details and broader contextual information.

Medical image segmentation, particularly for kidney and glomerulus structures, has attracted considerable research interest. Various studies have leveraged U-Net-based architectures with different backbone networks. For example, Singh Samant et al. (30) combined U-Net with ResNet18, ResNet50, InceptionV3, EfficientNetB1–B4, and VGG19 to improve glomerulus segmentation performance. Additionally, Gu et al. (31) proposed a multi-stream architecture based on FCN-ResNet and EfficientUNet, achieving an accuracy of up to 96.8%, while Shubham et al. (32) employed U-Net and EfficientNet, reporting an accuracy as high as 99.6%. More recently, Saikia et al. (33) introduced ResMLP-UNet, a novel segmentation architecture based on multilayer perceptrons (MLPs) rather than convolution or self-attention mechanisms, along with a customized MLP-UNet model. In Kaur et al. (1), U-Net was modified by incorporating additional convolutional blocks in both the encoder and decoder to learn features at multiple levels of detail, enabling effective localization of glomeruli in kidney images. For multiscale feature extraction, the Pyramid Pooling Module (PPM) (22) and Atrous Spatial Pyramid Pooling (ASPP) (7) were introduced, allowing models such as DeepLabv3+ to capture rich multiscale contextual information without significant computational overhead. Following these principles, CPP-UNet (34) combines PPM and ASPP pyramids to enhance feature discrimination across different scales for kidney, tumor, and cyst segmentation. Other pyramid pooling-based architectures, including Rema-Net (35), DS-UNeXt (28), and SEG-LUS (36), have also demonstrated strong performance in kidney segmentation tasks. Furthermore, DeepLab-based approaches (37), DeepLabv3+ (38), and DeepLabv3 (31) are widely adopted for precise kidney segmentation. To further address long-range dependency modeling and multiscale feature fusion, RWKVUNet was proposed in (39). This U-Net-based architecture integrates RWKV, GLSP, and CCM modules to capture long-range correlations, enhance multiscale feature aggregation, and improve medical image segmentation quality.

In summary, the LGC^2^-Former effectively captures both global contextual information and fine-grained local details by processing the input feature map through parallel global and local branches. By aggregating contextual cues along both horizontal and vertical directions, it enhances spatial awareness and significantly improves the segmentation accuracy of complex glomeruli structures.

### 2.2 Skip connection

Skip connections have been widely adopted in neural network architectures to address various challenges in deep learning. In particular, skip connections are employed to mitigate the loss of fine-grained spatial information, such as object boundary details, in encoder–decoder frameworks. U-Net (4) utilizes skip connections to transfer high-resolution feature maps from the encoder to the decoder, thereby improving boundary localization and segmentation accuracy. To further enhance representational capacity, U-Net++ (5) replaces simple skip connections with dense skip pathways, effectively reducing the semantic gap between encoder and decoder feature maps. Despite these advancements, both U-Net and U-Net++ rely on fixed receptive fields at each layer and intrinsic skip connection designs, which limit their ability to effectively model global and multiscale contextual information. This limitation is particularly pronounced in medical image analysis, where anatomical structures exhibit large variations in scale and appearance. Recent studies in medical image segmentation have highlighted the importance of skip connections in preserving fine details and improving boundary delineation. For instance, Edge-aware Skip Connections (ESC) integrate edge information into multi-level decoder features, facilitating hierarchical learning of discriminative representations for organ segmentation (40). CascadedVNet (41) modifies the conventional skip connection strategy by introducing block-wise connections between VNet modules and incorporating inception-like convolutions within the encoder to enrich feature representations. Similarly, the Hybrid CNN TransXNet (42) employs residual connections in both downsampling and upsampling stages to capture detailed local information and global contextual cues, leading to improved segmentation accuracy.

Recent contributions have also focused on scale-aware feature learning. For example, a 3D Scale-Aware Feature Extraction (SAFE) module utilizes adaptive kernel sizes to accommodate varying target scales, such as different organ and tumor sizes, without requiring fine-tuning. Furthermore, MultiResNet-SC enhances skip connections using ConvLSTM modules to preserve high-resolution details and capture spatial correlations in 3D images. Dense residual blocks, hybrid attention mechanisms, and multi-step upsampling with side-output deep supervision enable progressive learning of segmentation masks across multiple scales, although false positives may still persist (43). In encoder–decoder-based medical image segmentation pipelines, skip connections play a critical role in improving segmentation accuracy by allowing the decoder to directly access feature maps from corresponding encoder layers. This mechanism preserves fine spatial and structural details that would otherwise be lost during downsampling, resulting in more precise boundary localization.

Motivated by these observations, we redesign the skip connection mechanism by introducing the HMFA module, which promotes richer feature extraction and effective multi-scale fusion by enabling mutual guidance between consecutive layers, addressing the underutilization of complementary feature information.

### 2.3 Upsampling methods

Convolutional Neural Networks (CNNs) are well established and widely employed for dense prediction tasks. A critical operation in such networks is the upsampling of low-resolution feature maps, which is essential for visualization and the recovery of spatial information. Two primary approaches are commonly used for upsampling: interpolation-based methods and deconvolution (transposed convolution).Interpolation techniques, including nearest-neighbor, bilinear, and bicubic interpolation, are computationally efficient and simple to implement. However, these methods rely on predefined parameters, which often result in blurring and aliasing artifacts. In contrast, deconvolution employs learnable filters to increase spatial resolution during training, enabling feature-adaptive upsampling. Despite this advantage, uneven kernel overlap can cause certain regions to receive more contributions than others, leading to undesired checkerboard artifacts. Originally introduced in Fully Convolutional Networks (FCNs), deconvolution has since been widely adopted in segmentation architectures such as U-Net.To overcome these limitations, Shi et al. (44) proposed a learnable upsampling strategy known as Pixel Shuffle (PS), also referred to as sub-pixel convolution in the context of single-image super-resolution. The PS method effectively maps channel-wise feature representations into the spatial domain, providing a larger receptive field while preserving fine spatial details and minimizing distortion. As a result, PS is particularly well suited for high-quality segmentation tasks.Our experimental results indicate that conventional upsampling methods, such as deconvolution and bilinear interpolation, frequently introduce visual artifacts, especially checkerboard patterns, in the reconstructed images.

To address this issue, we incorporate Pixel Shuffle within the proposed Feature-Refined Upsampling (FRU) decoder module to enhance the spatial resolution of output feature maps while leveraging the multiscale feature extraction capability of the LGC^2^-Former. By reorganizing channel information into the spatial dimension, Pixel Shuffle enables artifact-free high-resolution reconstruction without the distortions commonly caused by interpolation-based methods (44). The integration of Pixel Shuffle not only alleviates edge blurring and foreground artifacts resulting from information loss but also improves the accuracy and robustness of the segmentation network.

While recent hybrid models such as TransUNet (45) and Swin-Unet (46) have successfully integrated Transformers into medical segmentation, they typically rely on sequential stacking of standard attention blocks and simple concatenation-based skip connections. In contrast, our LGC^2^-Former introduces a parallel dual-stream paradigm that decouples global context from local residuals before applying attention, addressing the specific challenge of preserving glomerular boundaries amidst stain variations. Furthermore, unlike the passive skip connections found in UNet++ (47) or the spatial gating in Attention U-Net (48), our HMFA module implements an active cross-layer query-key interaction, where shallow spatial features dynamically select relevant semantic contexts from deeper layers. Finally, we diverge from the standard use of deconvolution layers by adapting Pixel Shuffle (FRU) for segmentation, explicitly targeting the elimination of checkerboard artifacts common in existing encoder-decoder networks.

## 3 Method

**Figure 1:**
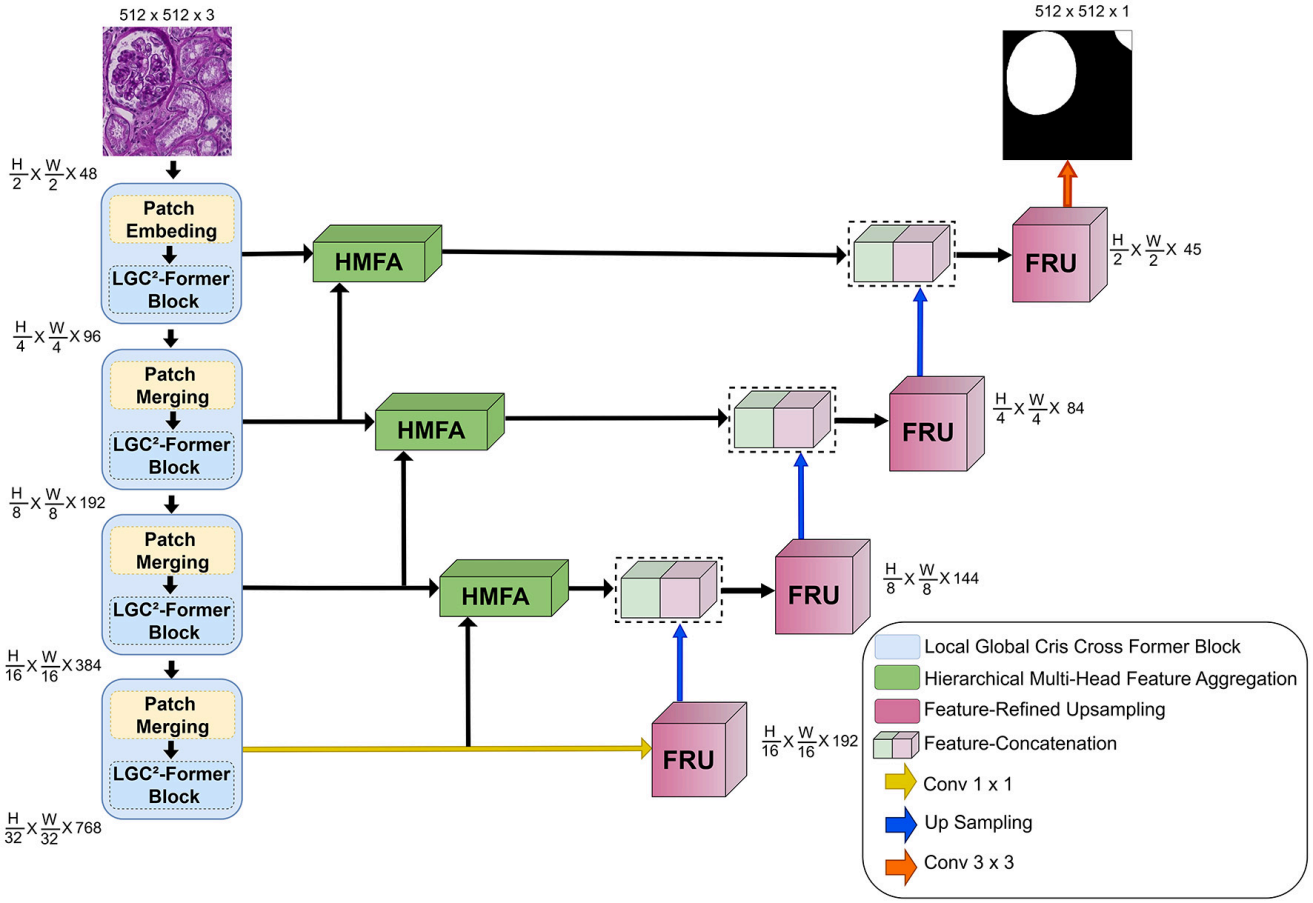
Architecture of GlomNet with the proposed LGC2-Former, HMFA and FRU modules.

GlomNet (Figure 1) is a glomeruli segmentation network designed for diverse staining protocols in whole-slide images (WSIs) for kidney disease diagnosis. It includes three components. The Local-Global Cris-Cross Former (LGC^2^-Former) splits the input feature map into parallel streams. One stream captures long-range dependencies in the global context, while the other preserves fine-grained glomerular features. These streams are fused by aggregating contextual information along horizontal and vertical directions. The Hierarchical Multi-head Feature Aggregation (HMFA) promotes feature extraction and multi-scale information fusion, addressing the underutilization of features between layers. The Feature-Refined Upsampling (FRU) module improves the resolution of feature maps while reducing artifacts. Together, these components allow GlomNet to capture details and contextual relationships, improving segmentation of glomeruli and renal structures in WSIs.

### 3.1 LGC^2^-Former

**Figure 2:**
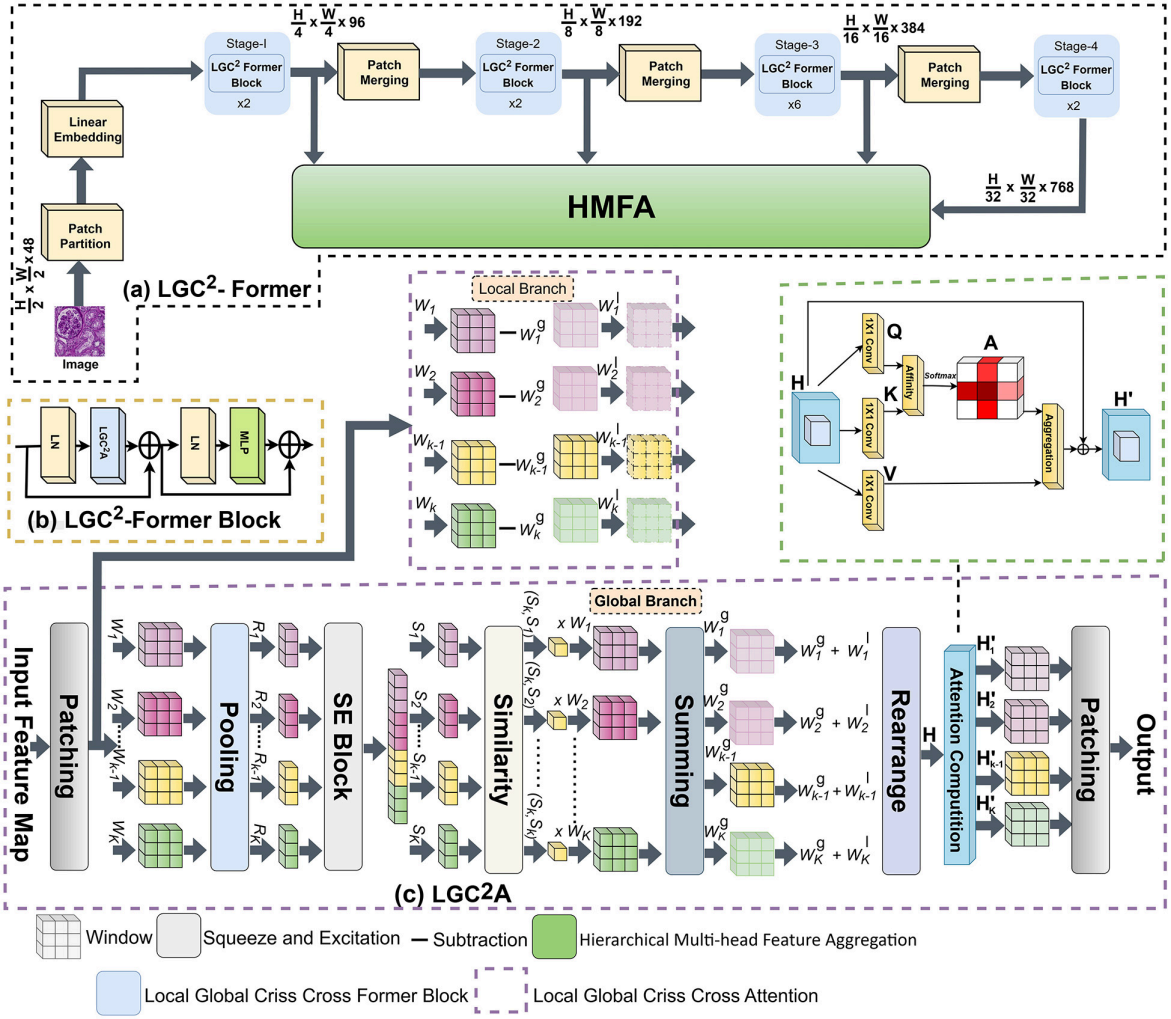
Architecture of LGC^2^-Former: **(a)** LGC^2^-Former at multiple stages of the encoder, **(b)** LGC^2^-Former block, **(c)** Local-Global Criss-Cross Attention.

Figure 2 presents the architecture of the LGC^2^-Former. The backbone network begins by splitting the input feature map into two parallel streams. The first stream, referred to as the global context branch, is responsible for learning long-range dependencies by analyzing row-wise feature distributions and measuring content-driven similarities between spatially distant but semantically related regions. This mechanism enables adaptive feature aggregation and allows the effective receptive field to expand dynamically according to learned similarity patterns, thereby capturing rich global contextual information. In parallel, the second stream, known as the local detail branch, emphasizes the preservation of fine-grained structural information. This is achieved by explicitly modeling the residual difference between the original local window features and the aggregated global context. Such a design ensures that discriminative local characteristics, particularly those associated with glomerular structures, are retained and not suppressed by dominant global representations. Following independent processing in the global and local branches, the resulting feature maps are fused and reorganized before being forwarded for attention computation. Attention mechanism aggregates contextual cues along both horizontal and vertical directions, enabling enhanced spatial awareness while maintaining structural continuity. By jointly leveraging global contextual reasoning and localized detail preservation, the LGC^2^-Former demonstrates strong capability in accurately segmenting complex renal structures, including glomeruli, across diverse histological staining modalities.

Let the input feature tensor be denoted as Equation 1:


$$\begin{array}{l} \text{X}\in {\mathbb{R}}^{H\times W\times C} \end{array}$$ (1)

It is partitioned into a set of non-overlapping windows of size *M*×*M*. Each window is represented as Equation 2:


$$\begin{array}{l} {\text{W}}_{k}\in {\mathbb{R}}^{M\times M\times C},\;k\in \left\{1,\ldots ,K\right\} \end{array}$$ (2)

where the total number of windows is Equation 3:


$$\begin{array}{l} K=\frac{H\times W}{M^{2}} \end{array}$$ (3)

To encode directional structural information within each window, the global branch computes weighted row-wise feature representations. This operation compresses the spatial complexity of each window from *M*^2^ elements to *M* elements while retaining essential horizontal patterns, which are particularly informative for glomerular morphology. The row-wise representation is defined as Equation 4:


$$\begin{array}{l} {\text{r}}_{k}=\frac{1}{M}\overset{M}{\underset{i=1}{\sum }}{\text{W}}_{k}\left(i,:,:\right)⊙\omega_{k} \end{array}$$ (4)

where ${\text{r}}_{k}\in {\mathbb{R}}^{M\times C}$, ⊙ denotes element-wise multiplication, and $\omega_{k}\in {\mathbb{R}}^{M\times C}$ is a learnable weighting parameter that allows the model to emphasize subtle but discriminative feature patterns within each window.

To further enhance feature discrimination, a Squeeze-and-Excitation (SE) module (49) is applied for channel-wise refinement as Equation 5:


$$\begin{array}{l} {\text{s}}_{k}=\text{SE}\left({\text{r}}_{k}\right) \end{array}$$ (5)

where ${\text{s}}_{k}\in {\mathbb{R}}^{C}$. The SE module assigns adaptive importance weights to each channel using a reduction ratio *r*, enabling the network to emphasize channels that contribute most to accurate glomeruli representation.

To model semantic relationships between spatially distant windows, content-based similarity is computed across all window pairs. Specifically, the similarity score between windows *k* and *j* is calculated using a dot-product operation as Equation 6:


$$\begin{array}{l} s_{k,j}=\frac{\exp\left(\frac{{\text{s}}_{k}{\text{s}}_{j}^{⊤}}{\tau }\right)}{\sum_{j=1}^{K}\exp\left(\frac{{\text{s}}_{k}{\text{s}}_{j}^{⊤}}{\tau }\right)} \end{array}$$ (6)

where τ is a temperature parameter controlling the sharpness of the similarity distribution. Smaller values of τ emphasize highly similar windows by sharpening the attention distribution, while larger values encourage broader contextual aggregation. In this work, τ is empirically selected via grid search within the range [0.01, 1.0] with an interval of 0.05. Through this optimization, τ = 0.1 was found to provide the most effective balance for cross-window contextual reasoning.

The resulting similarity map for window *k* is defined as Equation 7:


$$\begin{array}{l} {\text{S}}_{k}=\left[s_{k,1},s_{k,2},\ldots ,s_{k,K}\right]\in {\mathbb{R}}^{K} \end{array}$$ (7)

where values close to 1 indicate strong semantic similarity.

Using this similarity map, adaptive feature aggregation is performed to obtain the global representation of each window (Equation 8):


$$\begin{array}{l} {\text{W}}_{k}^{g}=\text{Linear}\left(\overset{K}{\underset{j=1}{\sum }}s_{k,j}{\text{W}}_{j}\right) \end{array}$$ (8)

where ${\text{W}}_{k}^{g}\in {\mathbb{R}}^{M\times M\times C}$. This operation allows each window to dynamically expand its receptive field based on learned semantic relevance rather than fixed spatial neighborhoods.

To complement global contextual modeling, a local branch is introduced to preserve fine-grained structural details that may be suppressed by dominant global features. Such local variations often contain critical boundary and texture cues necessary for precise glomeruli localization.

The local residual feature representation for window *k* is computed as Equation 9:


$$\begin{array}{l} {\text{W}}_{k}^{l}=\text{Linear}\left({\text{W}}_{k}-{\text{W}}_{k}^{g}\right) \end{array}$$ (9)

where ${\text{W}}_{k}^{l}\in {\mathbb{R}}^{M\times M\times C}$. This residual formulation explicitly separates local details from global context, yielding a more balanced and informative representation.

Finally, the global and local feature representations are fused and rearranged to form a unified feature map. This combined representation is further refined by aggregating contextual information along both horizontal and vertical directions (50). Through this integration of global semantic awareness and local structural sensitivity, the LGC^2^-Former effectively segments complex anatomical structures such as glomeruli across histopathological images with diverse staining protocols.

Given the combined feature map as Equation 10:


$$\begin{array}{l} \text{H}\in {\mathbb{R}}^{C\times W\times H} \end{array}$$ (10)

the Criss-Cross Attention (CCA) (50) first applies two separate convolutional layers with 1 × 1 kernels to generate the query and key feature maps, denoted as **Q** and **K**, respectively. Both feature maps are defined as Equation 11:


$$\begin{array}{l} \left\{\text{Q},\text{K}\right\}\in {\mathbb{R}}^{C^{\prime }\times W\times H} \end{array}$$ (11)

where *C*′ represents a reduced channel dimension introduced to decrease computational complexity while retaining essential semantic information.

Using **Q** and **K**, an attention affinity map is constructed (Equation 12):


$$\begin{array}{l} \text{A}\in {\mathbb{R}}^{\left(H+W-1\right)\times \left(W\times H\right)} \end{array}$$ (12)

For a given spatial position *u* in **Q**, the corresponding query feature vector is extracted as Equation 13:


$$\begin{array}{l} {\text{Q}}_{u}\in {\mathbb{R}}^{C^{\prime }} \end{array}$$ (13)

Simultaneously, a set of key feature vectors is formed by collecting features from **K** that lie along the same row and column as position *u*. The *i*-th element in this set is denoted as Equation 14 and proceed as Equation 15:


$$\begin{array}{l} \Omega_{u}\in {\mathbb{R}}^{\left(H+W-1\right)\times C^{\prime }} \end{array}$$ (14)

$$\begin{array}{l} \Omega_{i,u}\in {\mathbb{R}}^{C^{\prime }} \end{array}$$ (15)

The affinity between the query feature **Q**_*u*_ and each corresponding key feature **Ω**_*i, u*_ is computed using a dot-product operation as Equation 16:


$$\begin{array}{l} d_{i,u}={\text{Q}}_{u}\Omega_{i,u}^{⊤} \end{array}$$ (16)

This affinity computation measures the degree of correlation between features along the horizontal and vertical directions, enabling efficient long-range contextual interaction at the pixel level while maintaining spatial precision. Here, *d*_*i, u*_∈*D* represents the correlation strength between the query feature *Q*_*u*_ and the corresponding contextual feature Ω_*i, u*_, where *i*∈{1, …, *H*+*W*−1}. The correlation matrix *D*∈ℝ^(*H*+*W*−1) × (*W*×*H*)^ encodes these relationships across spatial locations. A softmax operation is then applied to *D* along the channel dimension to generate the normalized attention map *A*. To further adapt feature representations, an additional 1 × 1 convolution is applied to the input feature map *H*, producing the value feature map *V*∈ℝ^*C*×*W*×*H*^. For each spatial position *u* in *V*, a feature vector $V_{u}\in {\mathbb{R}}^{C}$ is obtained, along with a corresponding set $\Phi_{u}\in {\mathbb{R}}^{\left(H+W-1\right)\times C}$. The set Φ_*u*_ consists of feature vectors extracted from *V* that lie within the same row and column as position *u*. Contextual information is aggregated through a weighted summation process defined as Equation 17:


$$\begin{array}{l} H_{u}^{\prime }=\overset{H+W-1}{\underset{i=0}{\sum }}A_{i,u}\Phi_{i,u}+H_{u} \end{array}$$ (17)

where $H_{u}^{\prime }$ denotes the refined feature vector at spatial position *u* in the output feature map *H*′∈ℝ^*C*×*W*×*H*^, and *A*_*i, u*_ represents the attention weight corresponding to channel *i* at position *u*. The aggregated contextual features are combined with the original local representation *H*_*u*_ via a residual connection, enabling pixel-wise enhancement.

This mechanism allows the network to incorporate broad contextual dependencies while selectively emphasizing relevant spatial information through the learned attention map. As a result, the enriched feature representations exhibit improved robustness and discriminative power, making them particularly effective for semantic segmentation tasks.

Finally, the patches are integrated using a patch feature merging mechanism. In this process, the attention-enhanced features $H_{k}^{\prime }$ are subsequently concatenated across all windows and normalized to reconstruct the complete feature map *O*, as defined by Equation 18:


$$\begin{array}{ll} O & =\text{LN}\left(\text{Concat}{\left\{H_{k}^{\prime }\right\}}_{k=1}^{K}\right) \end{array}$$ (18)

where $H_{k}^{\prime }\in {\mathbb{R}}^{M^{2}\times d}$ denotes the enhanced features of the *k*-th window, $w_{o}\in {\mathbb{R}}^{d\times d}$ is a learnable projection matrix, and the set ${\left\{O_{k}\right\}}_{k=1}^{K}$ contains *K* windows of size *M*^2^×*d*.

The LGC^2^-Former architecture is constructed by replacing the standard W-MSA and SW-MSA modules in Swin Transformer blocks with the proposed LGC^2^-Former module, while preserving the remaining components of the network. Following the Swin-T configuration (27), the network processes features through *n* = 4 Transformer layers, with hyperparameters set to *C* = 96, layer depths of [2, 2, 6, 2], and attention heads of [3, 6, 12, 24]. Each DSA module is followed by a two-layer MLP with GELU activation, and all modules are preceded by LayerNorm (LN) layers. Residual connections are applied after each module to facilitate gradient flow and stable learning. To generate hierarchical feature representations, the number of tokens is progressively reduced via patch merging layers as the network depth increases. This strategy preserves content-aware feature projections throughout the architecture. By emphasizing content similarity rather than spatial proximity, LGC^2^-Former captures fine-grained feature relationships and achieves a more nuanced understanding of the feature space, requiring only minimal adjustments to the conventional window-based attention mechanism in Transformer blocks.

### 3.2 Hierarchical Multi-Head Feature Aggregation (HMFA) module

**Figure 3:**
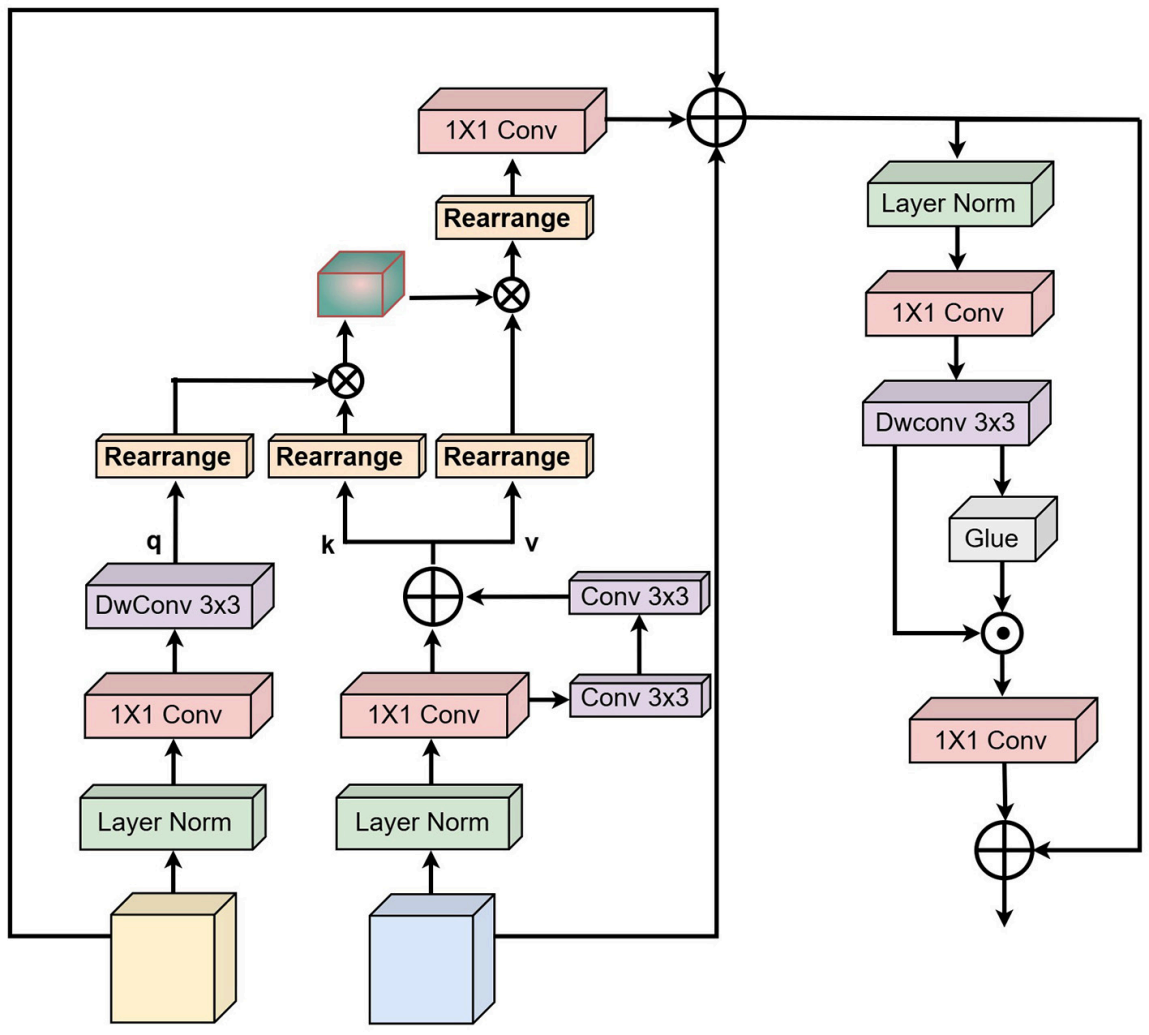
Architecture of HMFA with layer formation details.

To effectively segment structures such as glomeruli and handle irregular boundaries between glomeruli and surrounding tissue, we introduce a mechanism that allows mutual guidance between adjacent layers of the backbone network. This approach facilitates richer feature mining and the integration of multi-scale feature information. However, a naive combination of features from different layers often leads to redundancy and loss of discriminative information. Inspired by the Vision Transformer (26), we propose the Hierarchical Multi-Head Feature Aggregation (HMFA) module illustrated in Figure 3.

The HMFA module begins by performing layer normalization on feature maps extracted from two consecutive layers of the backbone network (shallow and deep). Let the normalized tensors be denoted as Equation 19 and Equation 20:


$$\begin{array}{l} X\in {\mathbb{R}}^{H\times W\times C} \end{array}$$ (19)

$$\begin{array}{l} Y\in {\mathbb{R}}^{H\times W\times C} \end{array}$$ (20)

where the query vector is generated from the shallow layer tensor *X*, while the key and value vectors are derived from the deep layer tensor *Y*. These vectors are further enriched with local contextual information.

Pixel-level cross-channel dependencies are first captured using a 1 × 1 convolution. Subsequently, two 3 × 3 depth-wise separable convolutions (DWC2D) encode channel-level spatial context over a larger receptive field. This combination ensures efficient feature extraction without significant computational overhead. The resulting query (Equation 21), key (Equation 22), and value (Equation 23) representations are formulated as:


$$\begin{array}{l} Q={\text{DWC2D}}_{3\times 3}\left({\text{DWC2D}}_{3\times 3}\left({\text{C2D}}_{1\times 1}\left(X\right)\right)\right) \end{array}$$ (21)

$$\begin{array}{l} K={\text{DWC2D}}_{3\times 3}\left({\text{DWC2D}}_{3\times 3}\left({\text{C2D}}_{1\times 1}\left(Y\right)\right)\right) \end{array}$$ (22)

$$\begin{array}{l} V={\text{DWC2D}}_{3\times 3}\left({\text{DWC2D}}_{3\times 3}\left({\text{C2D}}_{1\times 1}\left(Y\right)\right)\right) \end{array}$$ (23)

where C2D_1 × 1_(·) denotes a 1 × 1 convolution and DWC2D_3 × 3_(·) denotes a 3 × 3 depth-wise separable convolution. This structure enhances feature extraction while maintaining computational efficiency and allows the module to preserve both fine-grained details and high-level semantic information.

To enable effective interaction between features, the Query and Key tensors are first reshaped to allow their dot product to generate a transpose attention map of size ℝ^*C*×*C*^. The attention operation is formulated as Equation 24:


$$\begin{array}{l} \text{Attention}\left(Q^{\prime },K^{\prime },V^{\prime }\right)=V^{\prime }\cdot \text{Softmax}\left(\frac{K^{\prime }\cdot Q^{\prime }}{\beta }\right) \end{array}$$ (24)

where *Q*′, *K*′, *V*′∈ℝ^*H*×*W*×*C*^ are the reshaped tensors of the original feature maps. The parameter β is a learnable scaling factor that controls the magnitude of the dot product prior to the softmax normalization.

The output feature map *P*′ is then computed as Equation 25:


$$\begin{array}{l} P^{\prime }={\text{C2D}}_{1\times 1}\left(R\left(\text{Attention}\left(Q^{\prime },K^{\prime },V^{\prime }\right)\right)\right)+X+Y \end{array}$$ (25)

where *X* and *Y* are the shallow and deep layer feature maps, respectively, and *R* denotes the rearrangement operation that restores the attention output to the original spatial dimensions. This step ensures that global context from deeper layers and local details from shallower layers are effectively integrated to enhance segmentation performance.

Following attention, a feedforward network (FFN) is applied to refine pixel-level representations. The input feature map is first normalized to produce *Z*∈ℝ^*H*×*W*×*C*^. A 1 × 1 convolution expands channel dimensions, followed by a 3 × 3 depth-wise separable convolution to encode spatial context from neighboring pixels, facilitating learning of local structures important for glomeruli segmentation. To further improve information flow, a gating mechanism is applied. After the depth-wise separable convolution, the feature map is split along the channel dimension into two branches. One branch is passed through a GeLU activation and then element-wise multiplied with the other branch. Finally, a 1 × 1 convolution reduces the channels back to the original dimension. This process is expressed as Equation 26 and followed by Equation 27 and Equation 28:


$$\begin{array}{l} Z_{1}={\text{DWC2D}}_{3\times 3}\left({\text{C2D}}_{1\times 1}\left(Z\right)\right) \end{array}$$ (26)

$$\begin{array}{l} Z_{2}=\delta \left(Z_{1}\right)⊙Z_{1} \end{array}$$ (27)

$$\begin{array}{l} Z^{\prime }={\text{C2D}}_{1\times 1}\left(Z_{2}\right)+P^{\prime } \end{array}$$ (28)

where δ denotes the GeLU activation function, ⊙ represents element-wise multiplication, and *Z*′ is the resulting feature map. This mechanism enhances the network's focus on subtle details while complementing other feature levels, leveraging rich contextual information for accurate segmentation of complex structures such as glomeruli.

### 3.3 Feature-Refined Upsampling (FRU) module

The Pixel Shuffle (PS) technique (44), originally developed for Single-Image Super-Resolution (SISR), enables the reconstruction of high-resolution outputs from low-resolution inputs. It provides a parameter-efficient approach for upsampling, which is especially useful for semantic segmentation tasks involving large-scale medical images, such as glomeruli, under limited memory conditions. Building on this concept, we design the Feature-Refined Upsampling (FRU) module to exploit the multi-scale features generated by the four stages of LGC^2^-Former and further refined by the HMFA modules.

The FRU module performs upsampling by rearranging the pixels within the feature map, reducing the number of channels by a factor of *r*^2^, which substantially lowers the computational cost for subsequent convolutional operations. This pixel rearrangement follows a periodic shuffling scheme, converting channel information into spatial resolution without any loss. By preserving the complete feature information, FRU effectively mitigates edge blurring and artifacts commonly introduced by conventional upsampling methods. The periodic shuffling ensures that each output pixel is mapped directly from a specific input channel, maintaining the original feature characteristics and spatial relationships. This prevents smoothing effects often observed in interpolation-based upsampling, resulting in sharper boundaries and more accurate preservation of fine structural details critical for glomeruli segmentation.

Each FRU stage is strategically positioned to receive input from its corresponding HMFA layer, applying pixel shuffle upsampling followed by a 3 × 3 convolution to refine the spatial details. To generate dense-level predictions, high-level features from the final encoder stage (block 4) are progressively upsampled and fused with skip connections from the HMFA stages. As illustrated in Figure 2, FRU3 represents the first dedicated feature-refined upsampling stage in the decoder path. It serves as the bridge between the deepest bottleneck features and the hierarchical skip connections. Specifically, the feature map from HMFA3 (originating from the third encoder stage) undergoes pixel shuffle upsampling with a scaling factor *r* = 2. This transforms the feature map from $\text{X}\in {\mathbb{R}}^{\frac{H}{8}\times \frac{W}{8}\times C}$ to an upsampled map ${\text{X}}^{\prime }\in {\mathbb{R}}^{\frac{H}{4}\times \frac{W}{4}\times \frac{C}{4}}$ without any loss of information as Equation 29:


$${X^{\prime }}_{\text{de}}^{\left(i\right)}=\text{PS}({\text{HMFA}}_{i})$$ (29)

where ${X^{\prime }}_{\text{de}}^{\left(i\right)}\in {\mathbb{R}}^{2H\times 2W\times C/4}$ represents the intermediate upsampled feature map at decoder stage *i*.

Although pixel shuffle ensures lossless upsampling, a subsequent 3 × 3 convolution is applied to reduce aliasing, incorporate spatial context, and refine the features. This combination produces spatially coherent and segmentation-optimized feature maps. The final feature representation at decoder stage *i* is thus obtained as Equation 30:


$$X_{\text{de}}^{\left(i\right)}=W_{k=3}({X^{\prime }}_{\text{de}}^{\left(i\right)})$$ (30)

where *W*_*k* = 3_ denotes the 3 × 3 convolution operator applied to the upsampled features.

## 4 Experimental analysis and results

### 4.1 Datasets

#### 4.1.1 NEPTUNE

**Figure 4:**
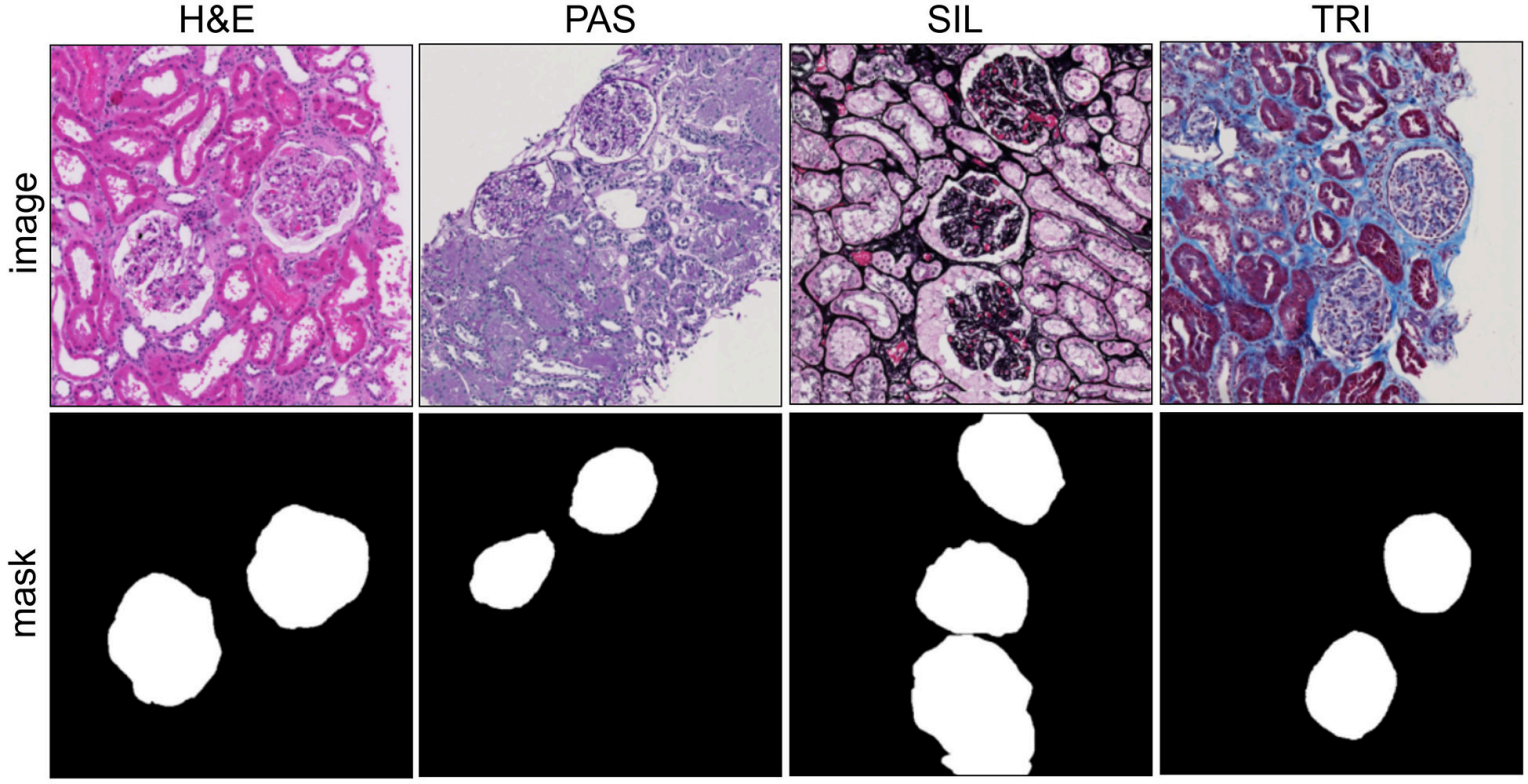
Images with corresponding masks taken from NEPTUNE.

The NEPTUNE dataset, also called the NEPTUNE subset (51), is derived from the multicenter Nephrotic Syndrome Study Network (NEPTUNE). It contains 620 publicly available whole-slide image (WSI) crops, referred to simply as WSIs. The annotations were curated by a panel of five nephropathologists. The dataset includes 125 biopsies of Minimal Change Disease (MCD) collected from 29 NEPTUNE centers, and 459 WSIs stained with various histological dyes including Hematoxylin & Eosin (H&E), Periodic Acid-Schiff (PAS), Silver (SIL), and Trichrome (TRI). Our work focuses on glomerular units, excluding other kidney structures such as glomerular tufts, tubules, and capillaries; while the raw dataset provided 615 images at 3, 000 × 3, 000 resolution, these were resized to 512 × 512 pixels for our experiments. Representative images with corresponding masks are shown in Figure 4, and the distribution of glomeruli across stains is summarized in Table 1.

**Table 1:** Glomerular annotations across staining techniques in the NEPTUNE subset.

Stain	Number of WSIs	Number of glomeruli
H&E	203	431
PAS	119	211
SIL	123	270
TRI	137	329

#### 4.1.2 HuBMAP - Hacking The Kidney (HuBMAP-1)

**Figure 5:**
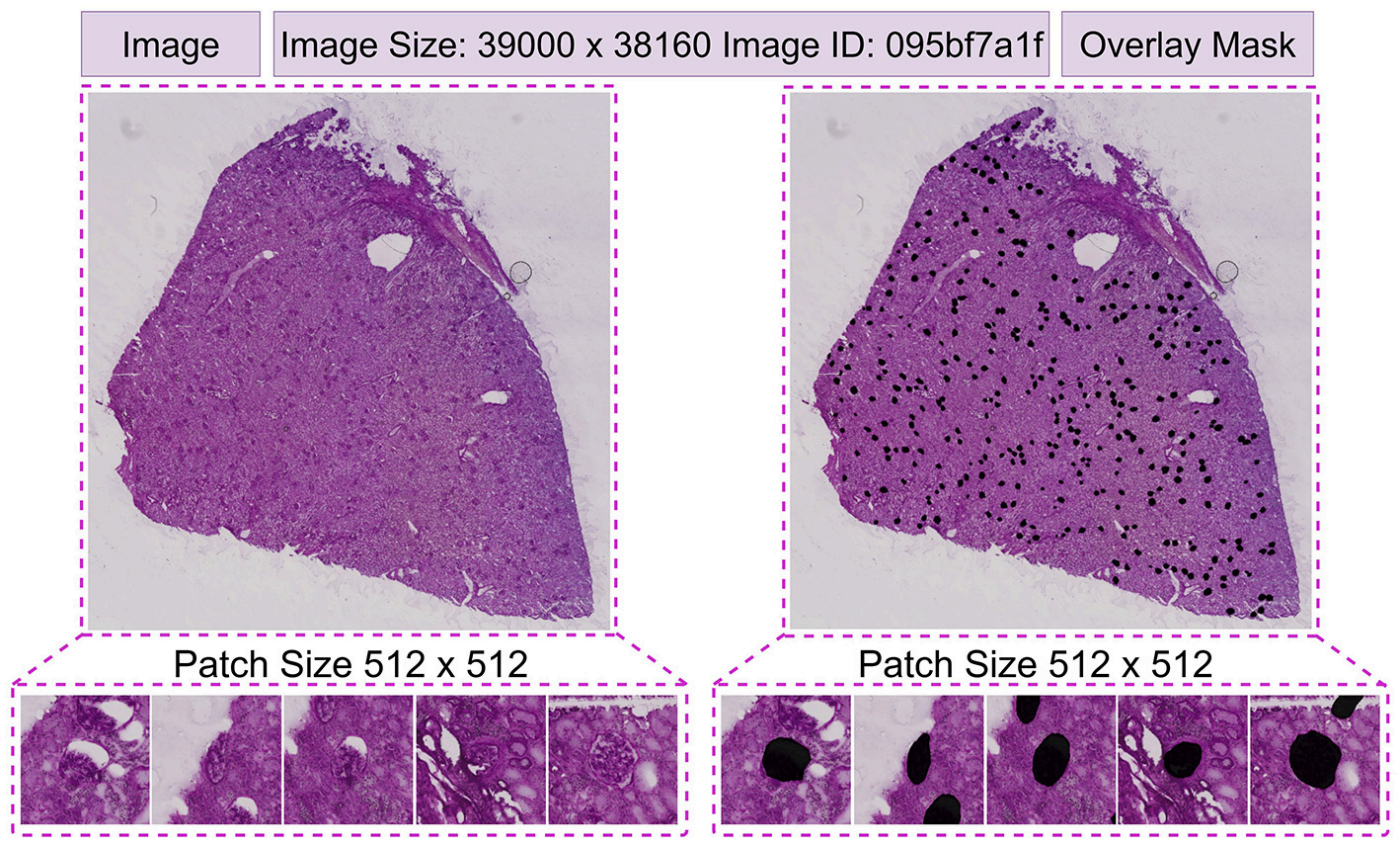
HuBMAP-1 image showing the original high-resolution image with an overlay mask and extracted patches.

The HuBMAP-1 dataset, funded by the National Institutes of Health (NIH), was originally released via a Kaggle competition and is now publicly accessible (52). Its goal is to map the human kidney's vascular system with a focus on functional tissue units (FTUs) known as glomeruli. The dataset includes 11 fresh frozen kidney images and 9 FFPE PAS-stained kidney samples, provided as high-resolution TIFF files (dimensions exceed 19,780 × 26,840 pixels, sizes from 182.65 MB to 4.87 GB). To ensure fair experimentation and robust generalization, we adopted a mixed-distribution strategy where patches for the training, validation, and testing sets were sampled across all available WSIs. Expert annotations for glomeruli are provided in both RLE-encoded and JSON formats. Table 2 summarizes the metadata, and example images with annotations are shown in Figure 5.

**Table 2:** HuBMAP-1 attributes and descriptions.

Attribute	Description
Width/height_pixels	Image dimensions in pixels
Patient_number	Identifier for tissue donor
Race, Ethnicity, Sex, Age	Demographic information of donors

#### 4.1.3 HuBMAP + HPA - Hacking the Human Body (HuBMAP-2)

**Figure 6:**
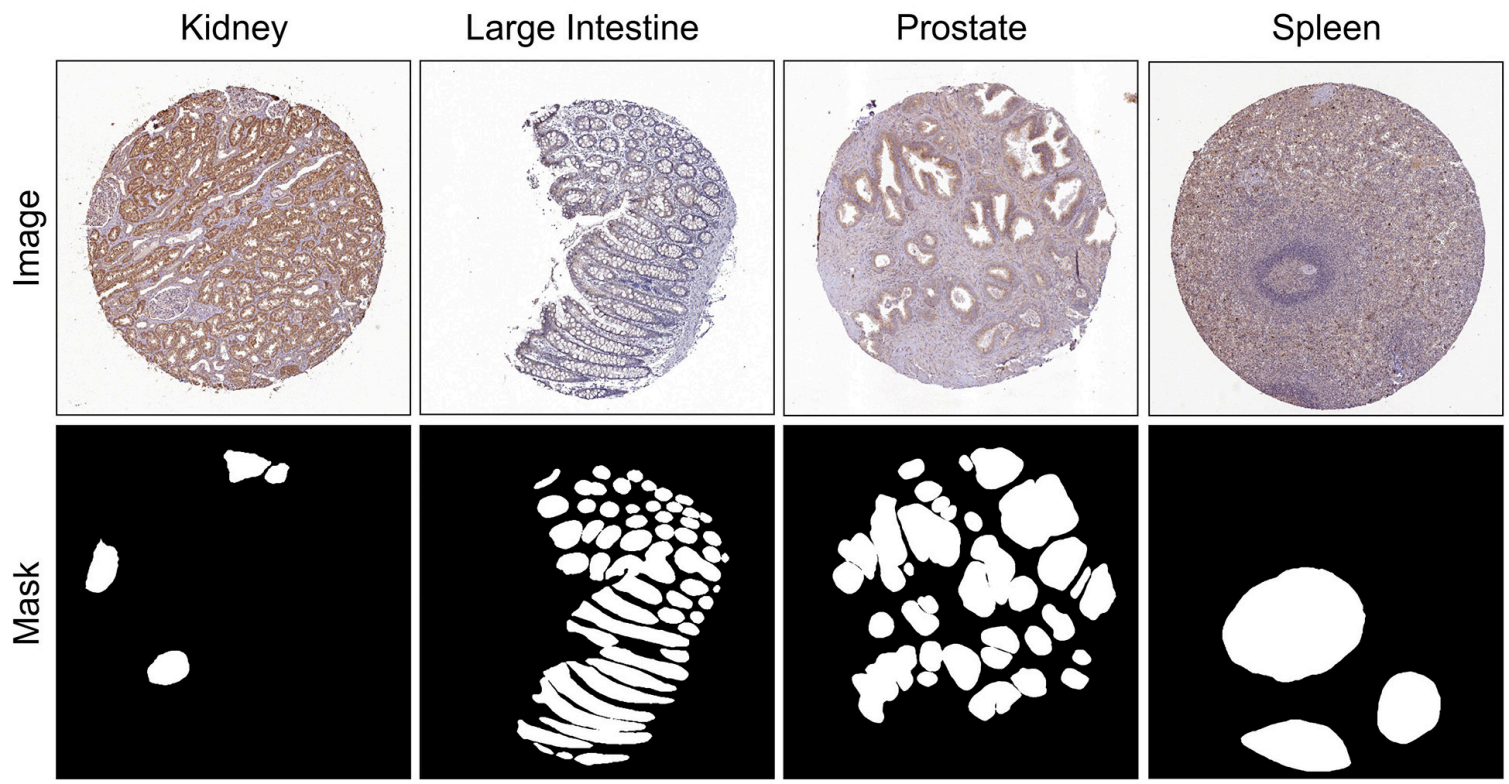
Images with corresponding masks taken from HuBMAP-2.

The HuBMAP-2 dataset combines FTU images from five human organs: kidney, large intestine, lung, prostate, and spleen. Publicly available via Kaggle, the dataset contains 351 training images of size 3, 000 × 3, 000 pixels with corresponding masks and FTU labels. Images originate from two sources: HuBMAP and the Human Protein Atlas (HPA), resulting in technical variability. HPA images have uniform specifications (pixel size 0.4 μm, slice thickness 4 μm), while HuBMAP images vary in pixel size (0.229–6.263 μm) and slice thickness (4–10 μm). Figure 6 shows example images with masks, and Table 3 summarizes dataset attributes.

**Table 3:** HuBMAP-2 attributes and descriptions.

Attribute	Description
Id	Image identifier
Organ	Organ type: kidney, prostate, lung, spleen, large intestine
img_height	Image height (pixels)
img_width	Image width (pixels)
pixel_size	Physical size of one pixel (μm)
RLE	Run-length encoding of FTU annotations
Age	Donor's age (years)
Sex	Donor's sex

### 4.2 Implementation details

All experiments were conducted using the PyTorch framework on an NVIDIA RTX 3090 GPU. The segmentation model is based on a Swin-T backbone integrated with a UNet architecture. The model was trained with a batch size of 4 for 200 epochs on the NEPTUNE dataset and 150 epochs on the HuBMAP-1 and HuBMAP-2 datasets. The initial learning rate was set to 1 × 10^−3^, and the Adam optimizer (53) was employed with a weight decay of 2 × 10^−4^. A stepwise learning rate decay was applied, reducing the rate by a factor of 0.85 every 10 epochs for NEPTUNE, and every 15 epochs for HuBMAP-1 and HuBMAP-2. This learning rate schedule was chosen after empirical optimization for the best performance. To ensure a fair comparison, this optimized configuration was applied consistently across all SOTA comparative models rather than using disparate default settings. To assess performance, standard metrics were used: precision (Pre), recall (Rec), F1-score (F1), intersection over union (IoU) and HD95 (Hausdorff Distance at the 95th percentile). Each dataset was split into 70% training, 20% validation, and 10% testing, ensuring an independent test set. To enhance robustness and generalization, data augmentation including flipping, rotation, and shearing was applied.

### 4.3 Ablation studies

#### 4.3.1 Comparison of encoder variants

To investigate the influence of different backbone architectures within GlomNet, we performed an ablation study on the HuBMAP-1 dataset. Table 4 compares LGC^2^-Former against standard backbones: ResNet50, Swin-T, and ViT. The results demonstrate that LGC^2^-Former outperforms all other backbones across all metrics, achieving 97.23% precision, 94.95% recall, 96.09% F1-score, and 92.46% IoU. ResNet50, while effective for general image tasks, achieves only 87.31% F1-score, highlighting its limitations in capturing fine glomerular details. Swin-T, a transformer-based backbone, shows improved performance (89.72% F1) by modeling long-range dependencies, but it still falls short of LGC^2^-Former due to less effective local-global feature integration. ViT improves further (92.18% F1) but does not reach the specialized capability of LGC^2^-Former, which combines feature aggregation with targeted attention for precise glomeruli segmentation.

**Table 4:** Comparison of LGC^2^-Former with different backbones on HuBMAP-1.

Backbone	Pre (%)	Rec (%)	F1 (%)	IoU (%)	Params (M)	FLOPs (G)
ResNet50 (54)	88.78	85.88	87.31	77.48	61.85	**13.7**
Swin-T (55)	90.56	88.92	89.72	81.35	65.32	186.38
ViT (26)	93.32	91.09	92.18	85.51	90.56	103.89
LGC^2^-Former	**97.23**	**94.95**	**96.09**	**92.46**	**48.12**	185.72

Bold values indicate the highest achieved values. Underline values indicate the 2nd highest achieved values.

#### 4.3.2 Effectiveness of LGC^2^-Former, HMFA, and FRU

To quantify the contribution of each module in GlomNet, we conducted ablation experiments on HuBMAP-1. Table 5 presents performance with different combinations of LGC^2^-Former, HMFA, and FRU, with UNet + Swin-T as the baseline. Baseline (no modules): Pre 90.56%, Rec 88.92%, F1 89.72%, IoU 81.35%. LGC^2^-Former only: Pre 94.50%, Rec 91.53%, F1 92.98%, IoU 86.88% - showing the importance of capturing both local and global features. HMFA only: Pre 92.53%, Rec 90.35%, F1 91.43%, IoU 84.21% improving layer-wise feature aggregation. FRU only: Pre 90.87%, Rec 89.80%, F1 90.32%, IoU 82.37% refining upsampling for better segmentation detail. LGC^2^-Former + FRU: Pre 94.88%, Rec 92.95%, F1 93.90%, IoU 88.51% combining local-global features with refined upsampling. All modules enabled: Pre 97.23%, Rec 94.95%, F1 96.09%, IoU 92.46% achieving optimal segmentation performance. This study confirms that LGC^2^-Former contributes the most, followed by FRU and HMFA, with maximum accuracy achieved when all modules are combined.

**Table 5:** Ablation study: contributions of LGC^2^-Former, HMFA, and FRU on HuBMAP-1.

LGC^2^-Former	HMFA	FRU	Pre (%)	Rec (%)	F1 (%)	IoU (%)	Params (M)	FLOPs (G)
×	×	×	90.56	88.92	89.72	81.35	27.17	-
✓	×	×	94.50	91.53	92.98	86.88	41.20	179.26
×	✓	×	92.53	90.35	91.43	84.21	28.29	10.36
✓	✓	×	94.17	92.05	93.11	87.06	42.32	180.62
×	×	✓	90.87	89.80	90.32	82.37	32.97	15.10
✓	×	✓	94.88	92.95	93.90	88.51	47.00	184.36
×	✓	✓	92.85	91.50	92.17	85.49	34.09	16.46
✓	✓	✓	**97.23**	**94.95**	**96.09**	**92.46**	**48.12**	**185.72**

Bold values indicate the highest achieved values.

#### 4.3.3 Comparison of decoder variants

Deconvolution and bilinear interpolation are widely used upsampling methods to restore spatial details lost during convolution or max-pooling operations. Unlike these traditional methods, FRU is built upon subpixel convolution, which more effectively addresses issues like blurry edges and checkerboard artifacts. To evaluate the impact of various upsampling methods on the performance of LGC^2^-Former with HMFA, we conducted an ablation study using the HuBMAP-1 dataset. Table 6 compares the performance of DeConvolution, Bilinear, and FRU as upsampling techniques, all in conjunction with LGC^2^-Former and HMFA. The results show that the FRU upsampling method achieves the best performance across all metrics, with a precision of 97.23%, recall of 94.95%, F1 score of 96.09%, and IoU of 92.46%. This demonstrates the superior ability of FRU to capture the fine details necessary for precise glomeruli segmentation, leveraging both local and global feature representations. When DeConvolution is used as the upsampling method, the model performs with precision of 90.56%, recall of 88.92%, F1 score of 89.72%, and IoU of 81.35%. While DeConvolution is a widely used technique for upsampling, its performance is lower compared to FRU, indicating that it struggles to capture finer details as effectively. Similarly, when Bilinear interpolation is used, the performance improves over DeConvolution, with precision of 95.79%, recall of 90.08%, F1 score of 90.84%, and IoU of 83.25%. While Bilinear interpolation helps to recover spatial information, it still doesn't match the accuracy and detail preservation offered by FRU.

**Table 6:** Effectiveness of different UpSampling methods on HuBMAP-1.

LGC^2^-Former	HMFA	UpSampling	Pre (%)	Rec (%)	F1 (%)	IoU (%)	HD95 (mm)
✓	✓	DeConvolution	90.56	88.92	89.72	81.35	18.15
✓	✓	Bilinear	95.79	90.08	90.84	83.25	16.59
✓	✓	FRU	97.23	94.95	96.09	92.46	9.53

Bold values indicate the highest achieved values.

**Figure 7:**
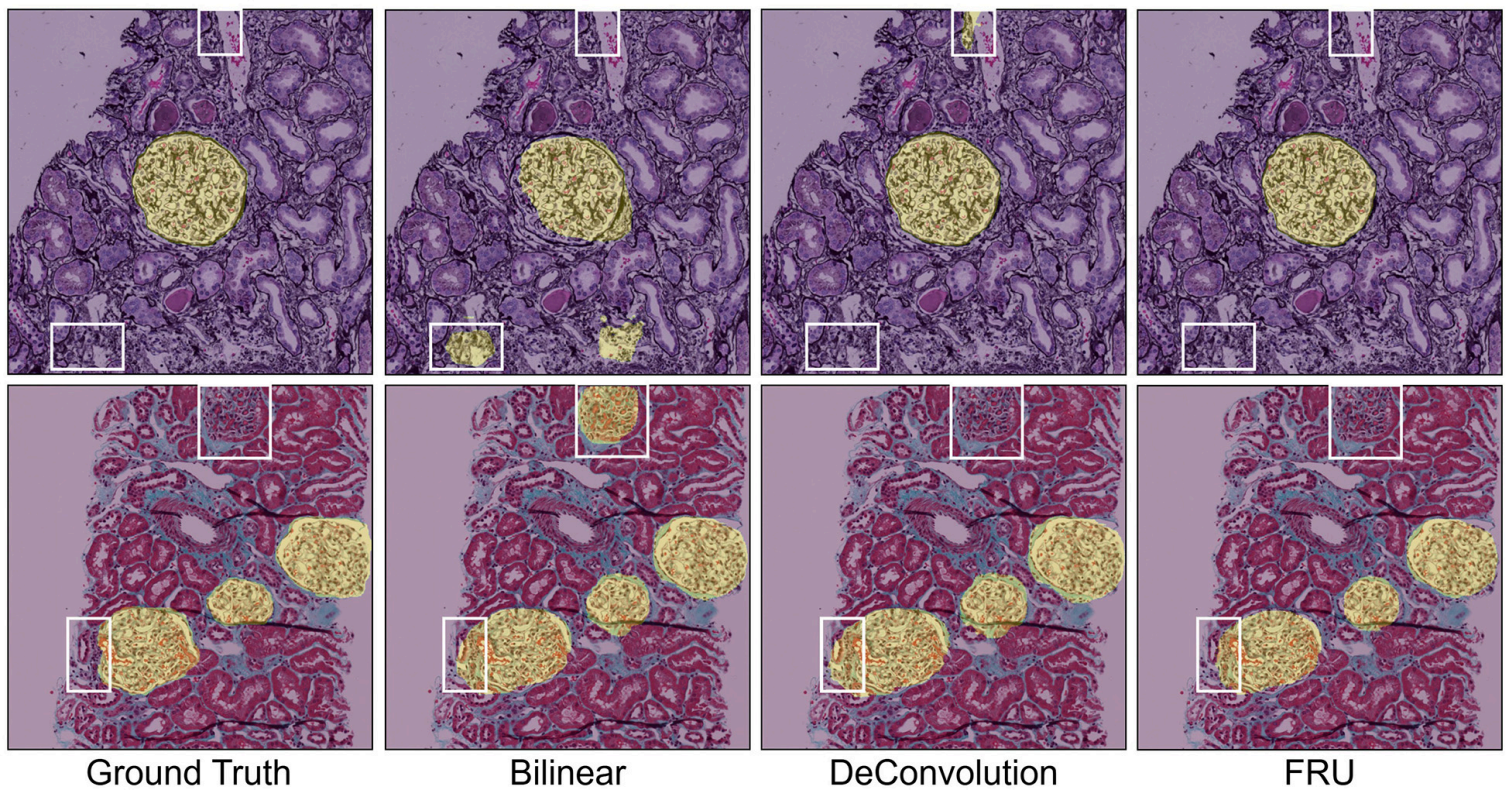
Qualitative comparisons of GlomNet with deconvolution, bilinear, and FRU-based upsampling methods.

The qualitative comparisons of GlomNet with traditional upsampling methods such as deconvolution, bilinear, and FRU based upsampling are presented in Figure 7. From left to right, the rows showcase results on the NEPTUNE test dataset, highlighting the performance differences and the enhanced capabilities of GlomNet in comparison to the other methods.

### 4.4 Comparison with SOTA methods

#### 4.4.1 Results on NEPTUNE

This section presents the evaluation of GlomNet on the NEPTUNE dataset, comparing it with state-of-the-art (SOTA) methods including UNet, RWKV-UNet, SelfReg-UNet, and CNN-TransXNet. Table 7 summarizes the quantitative results for different staining types: PAS, H&E, SIL, and TRI. Across all stains, GlomNet consistently outperforms competing methods in precision, recall, F1 score, and IoU, demonstrating its ability to capture fine details and complex contextual information. For PAS staining, GlomNet achieves 94.37% precision, 91.06% recall, 92.68% F1 score, and 86.37% IoU, reflecting accurate detection of glomerular structures in texture-rich regions. RWKV-UNet follows closely with slightly lower metrics, while SelfReg-UNet and CNN-TransXNet exhibit lower performance due to less effective contextual aggregation. For H&E staining, which is challenging due to low contrast and subtle textural differences, GlomNet achieves 84.70% precision, 82.99% recall, 83.83% F1 score, and 72.17% IoU, surpassing all other methods. The results highlight GlomNet's ability to maintain segmentation accuracy even under low inter-class variance conditions. For SIL staining, characterized by strong gradients at boundaries, GlomNet achieves 89.09% precision, 86.83% recall, 87.94% F1 score, and 78.49% IoU. While RWKV-UNet and SelfReg-UNet also perform reasonably well, GlomNet's combination of LGC^2^-Former and HMFA ensures superior segmentation along intricate boundaries. TRI staining introduces a multimodal challenge characterized by significant intra-class variability. Despite these complexities, our model achieves 84.34% precision, 82.78% recall, 83.55% F1-score, and 71.75% IoU. It outperforms competing methods by successfully integrating local structural details with global contextual information to address the diverse visual appearances of the glomerular units.

**Table 7:** GlomNet qualitative comparison with SOTA methods on NEPTUNE.

Stain	Methods	Pre (%)	Rec (%)	F1 (%)	IoU (%)
PAS	UNet (4)	88.18	84.19	86.13	75.65
	RWKV-UNet (39)	93.59	90.35	91.94	85.09
	SelfReg-UNet (56)	91.38	90.96	91.17	83.78
	CNN-TransXNet (42)	91.33	89.87	90.59	82.77
	GlomNet	**94.37**	**91.06**	**92.68**	**96.37**
H&E	UNet (4)	78.56	73.55	75.97	61.23
	RWKV-UNet (39)	84.21	80.89	82.51	70.24
	SelfReg-UNet (56)	82.90	80.13	81.49	68.76
	CNN-TransXNet (42)	81.20	79.78	80.48	67.34
	GlomNet	**84.70**	**82.99**	**83.83**	**72.17**
SIL	UNet (4)	81.53	79.22	80.35	67.16
	RWKV-UNet (39)	**89.10**	85.56	87.29	77.46
	SelfReg-UNet (56)	87.99	85.49	86.72	76.56
	CNN-TransXNet (42)	86.69	84.98	85.83	75.16
	GlomNet	89.09	**86.83**	**87.94**	**78.49**
TRI	UNet (4)	77.79	76.71	77.25	62.93
	RWKV-UNet (39)	**84.70**	81.21	82.91	70.82
	SelfReg-UNet (56)	83.28	81.25	82.25	69.86
	CNN-TransXNet (42)	81.95	80.64	81.29	68.48
	GlomNet	84.34	**82.78**	**83.55**	**71.75**

Bold values indicate the highest achieved values. Underline values indicate the 2nd highest achieved values.

**Figure 8:**
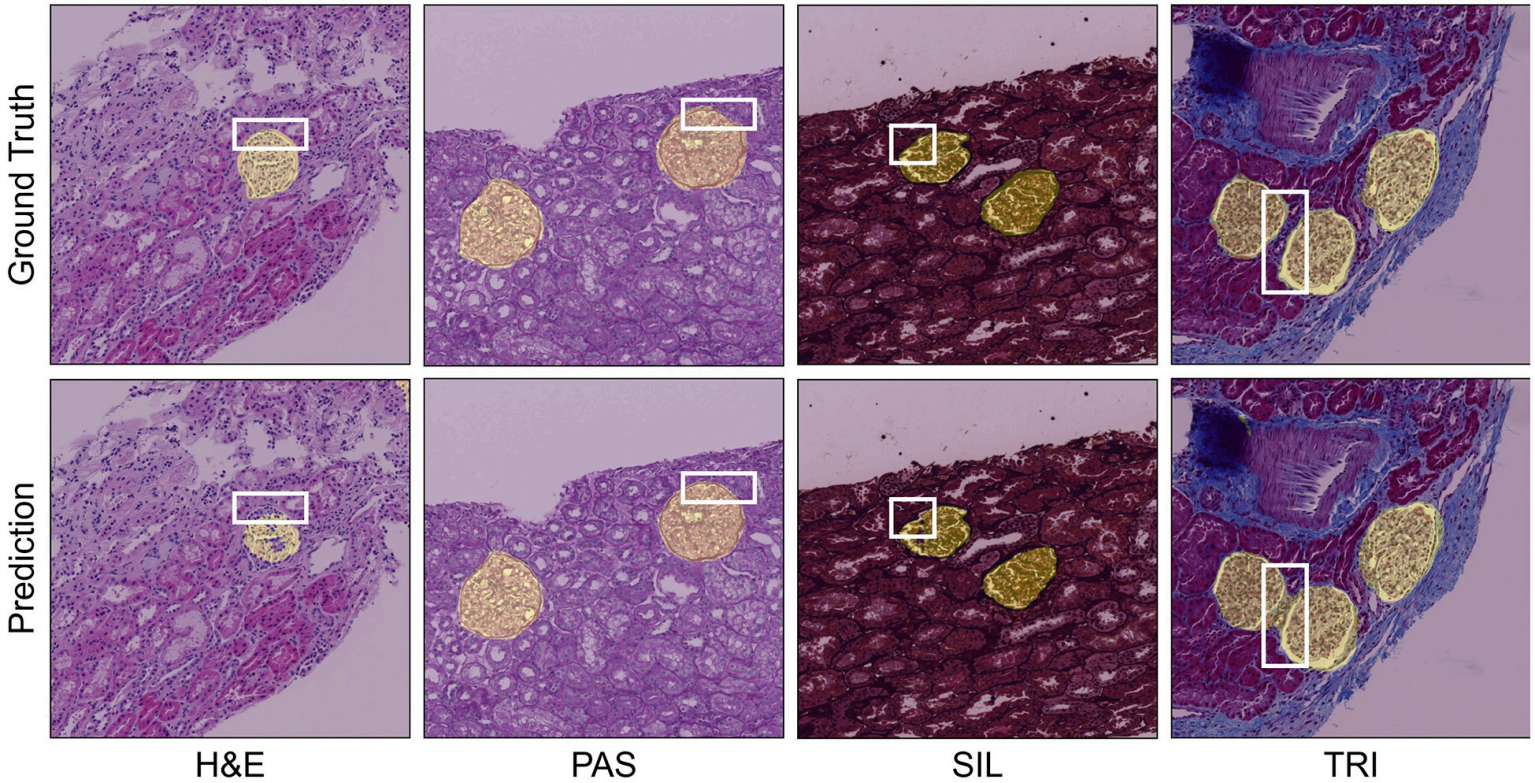
GlomNet qualitative cross-validation on NEPTUNE for H&E, PAS, SIL, and TRI staining.

The model was trained on the HuBMAP-1 dataset using PAS images and then cross-validated on the NEPTUNE dataset for multi-stain segmentation (Figure 8). In the case of H&E staining, the model effectively identifies the region of interest; however, it occasionally misses certain portions of the region. For PAS staining, the model performs exceptionally well, accurately segmenting the relevant areas. When applied to SIL staining, it correctly segments regions corresponding to the glomeruli, demonstrating a good understanding of the tissue structures. However, for TRI staining, the model generates some false positives, incorrectly identifying regions that do not correspond to the glomeruli. This highlights the need for further refinement to improve TRI stain segmentation accuracy.

**Figure 9:**
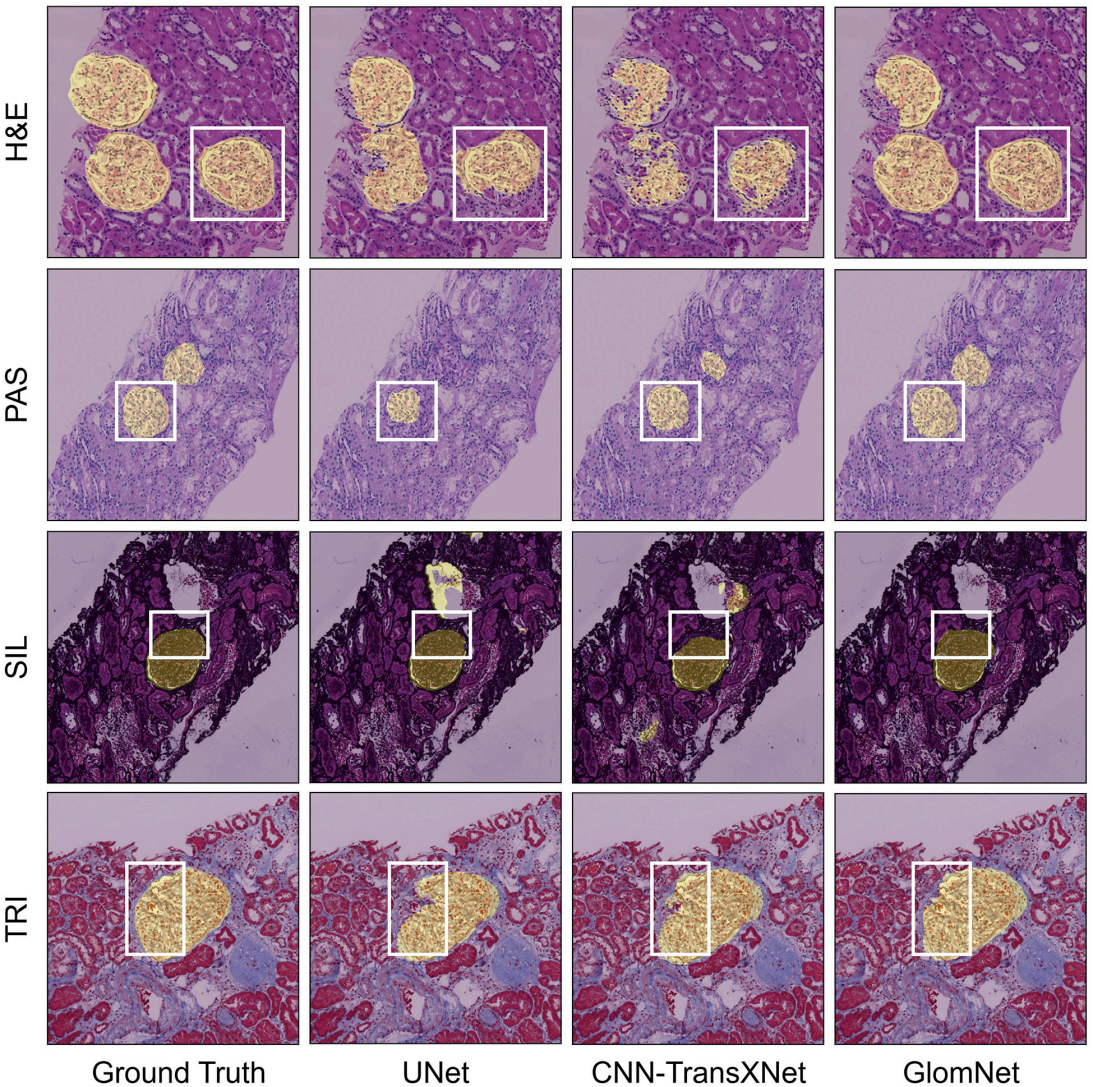
GlomNet qualitative comparison of NEPTUNE for H&E, PAS, SIL, and TRI staining.

Figure 9 provides a visual comparison of segmentation performance across the different staining types. For H&E, UNet fails to segment multiple glomeruli completely, while CNN-TransXNet reduces false positives but does not achieve full segmentation. GlomNet delivers the most accurate delineation. For PAS, UNet under-segments glomeruli, and CNN-TransXNet misses finer structures, whereas GlomNet achieves complete segmentation. For SIL, all methods capture major structures, but UNet and CNN-TransXNet still produce false positives; GlomNet shows minimal errors. For TRI, a complex multi-modal stain, GlomNet outperforms competitors by capturing detailed textures and global context simultaneously.

#### 4.4.2 Performance comparison with state-of-the-art methods on HuBMAP-1

This subsection presents a comprehensive evaluation of the proposed GlomNet on the HuBMAP-1 dataset, with a particular focus on PAS-stained WSIs. The performance of GlomNet is systematically compared with several state-of-the-art (SOTA) segmentation approaches to demonstrate its effectiveness in glomeruli segmentation. To ensure a fair and thorough comparison, quantitative results are reported using standard evaluation metrics, while qualitative results are also analyzed through visual inspection. Table 8 provides a direct comparison between GlomNet and recent SOTA models, including UNet, RWKV-UNet, SelfReg-UNet, and CNN-TransXNet. Among all competing methods, GlomNet achieves the best performance across all evaluation metrics. Specifically, it attains a precision of 97.23%, recall of 94.95%, F1 score of 96.09%, and IoU of 92.46%. These results indicate that GlomNet is highly effective in capturing both fine-grained structural details and long-range contextual information, which are critical for accurate glomeruli segmentation in PAS-stained tissue images. While RWKV-UNet and SelfReg-UNet also demonstrate strong performance, their results remain slightly inferior to GlomNet, particularly in terms of F1 score and IoU. In contrast, the conventional UNet exhibits noticeably lower performance, especially at object boundaries, highlighting its limited capability in modeling complex spatial dependencies. CNN-TransXNet improves upon UNet by integrating transformer-based representations; however, it still falls short of GlomNet, emphasizing the advantage of the proposed architecture in balancing local and global feature learning.

**Table 8:** Comparison with SOTA WSI on HuBMAP-1.

Methods	Pre (%)	Rec (%)	F1 (%)	IoU (%)
UNet (4)	90.56	88.92	89.72	81.35
TransUNet (45)	94.38	92.52	93.42	87.65
Swin-UNet (46)	94.96	92.71	93.61	87.99
CSWin-UNet (57)	95.22	93.31	94.14	88.93
RWKV-UNet (39)	96.38	93.97	95.17	90.77
SelfReg-UNet (56)	95.81	93.69	94.74	90.02
CNN-TransXNet (42)	96.69	91.23	93.88	88.46
GlomNet	**97.23**	**94.95**	**96.09**	**92.46**

Bold values indicate the highest achieved values.

**Figure 10:**
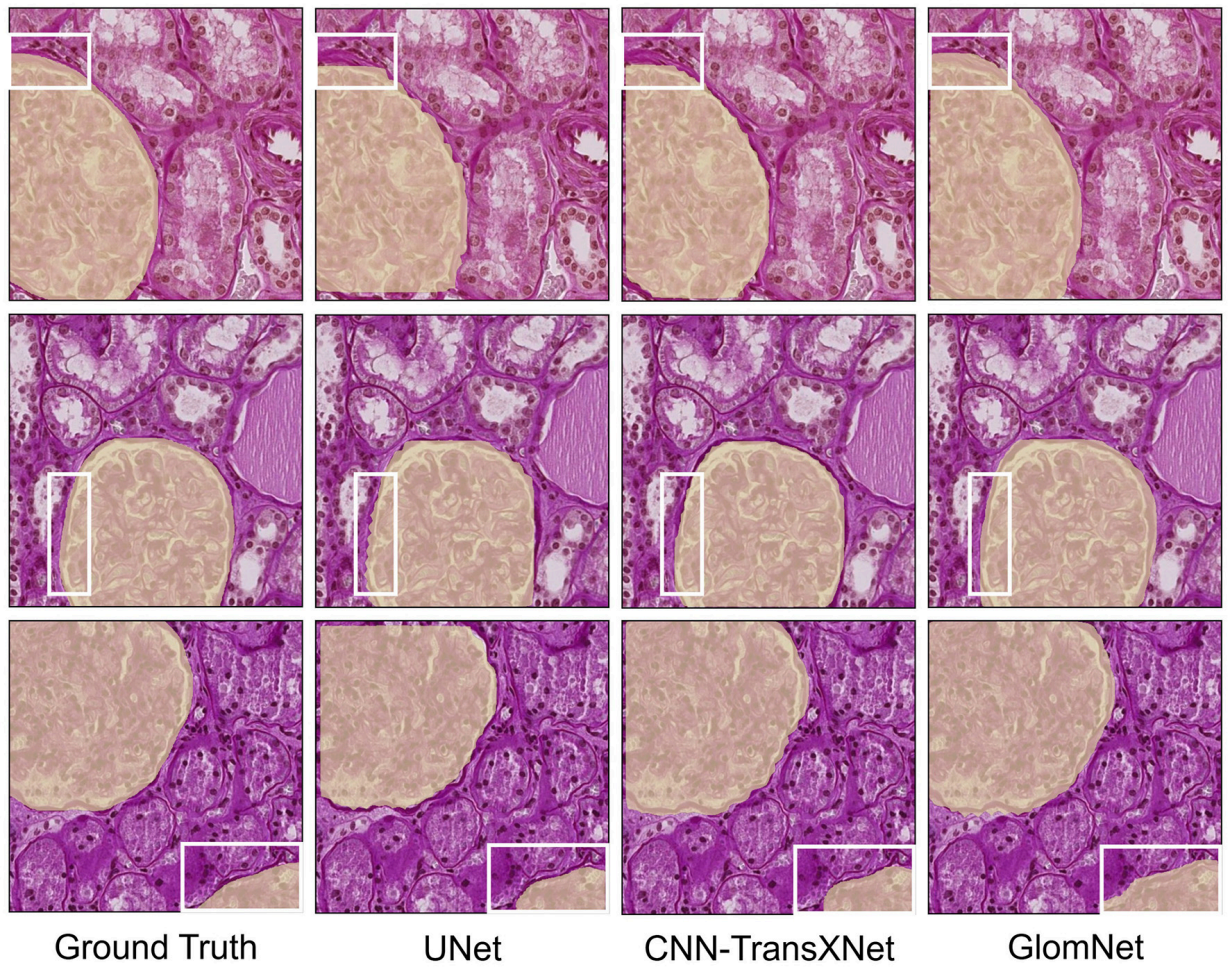
GlomNet qualitative comparison on HuBMAP-1.

To further validate the effectiveness of GlomNet, Table 9 compares its performance with previously published WSI segmentation methods on the HuBMAP-1 dataset. This comparison includes approaches such as EnsembleDLNet, FResMRCNN, InFeNet, DS-FNet, LinkNet, and SegNeXt. GlomNet achieves the highest F1 score of 96.09%, outperforming all listed methods. Notably, it surpasses InFeNet and SegNeXt, which are among the strongest existing approaches, thereby establishing GlomNet as a new state-of-the-art solution for glomeruli segmentation on the HuBMAP-1 dataset. In addition to quantitative evaluation, qualitative results are illustrated in Figure 10. The visualization comparison reveals clear differences among the models. UNet produces fragmented segmentation maps with inaccurate boundaries and frequent misclassification of foreground pixels as background. CNN-TransXNet improves boundary consistency and reduces false positives but still fails to generate complete and coherent segmentation masks. In contrast, GlomNet delivers the most precise and visually consistent segmentation results, accurately delineating glomerular structures with well-defined boundaries. These visual outcomes further confirmthe robustness and superiority of GlomNet in handling complex WSI segmentation tasks.

**Table 9:** Comparison of SOTA methods for WSI imagery on HuBMAP-1.

Model	Backbone	F1 (%)
EnsembleDLNet (31)	EfficientNet	91.51
FResMRCNN (58)	ResNet101	94.83
InFeNet (8)	ResNet50-RFE	95.57
DS-FNet (59)	EfficientNet	95.05
LinkNet (30)	EfficientNet	94.26
SegNeXt (60)	MSCAN-S	95.33
GlomNet	LGC^2^-Former	**96.09**

Bold values indicate the highest achieved values. Underline values indicate the 2nd highest achieved values.

#### 4.4.3 Performance evaluation with state-of-the-art methods on HuBMAP-2

This subsection evaluates the performance of the proposed GlomNet on the HuBMAP-2 dataset, which consists of H&E stained WSIs collected from multiple organs. Compared to PAS-stained images, H&E staining presents additional challenges due to subtle texture variations, low contrast between foreground and background regions, and complex tissue morphology. To demonstrate the robustness of GlomNet under these challenging conditions, its performance is compared with several state-of-the-art (SOTA) segmentation models. Table 10 presents a quantitative comparison between GlomNet and existing SOTA methods, including UNet, RWKV-UNet, SelfReg-UNet, and CNN-TransXNet. Among all evaluated approaches, GlomNet consistently achieves the highest scores across all evaluation metrics, recording a precision of 87.69%, recall of 85.81%, F1 score of 86.74%, and IoU of 76.60%. These results indicate that GlomNet is particularly effective in handling the complex appearance variations and ambiguous boundaries commonly observed in H&E-stained tissue samples.

**Table 10:** Comparison with SOTA WSI on HuBMAP-2.

Methods	Pre (%)	Rec (%)	F1 (%)	IoU (%)
UNet (4)	79.73	78.49	79.11	65.41
RWKV-UNet (39)	87.09	85.11	86.08	75.57
SelfReg-UNet (56)	86.43	83.87	85.12	74.14
CNN-TransXNet (42)	86.24	82.84	84.52	73.19
GlomNet	**87.69**	**85.81**	**86.74**	**76.60**

Bold values indicate the highest achieved values. Underline values indicate the 2nd highest achieved values.

RWKV-UNet demonstrates competitive performance, achieving an F1 score of 86.08% and IoU of 75.57%, which highlights the effectiveness of RWKV-based architectures in modeling long-range contextual dependencies. SelfReg-UNet and CNN-TransXNet also show reasonable segmentation accuracy; however, their performance remains inferior to GlomNet, especially in terms of IoU and F1 score. This performance gap suggests that GlomNet is more capable of preserving fine structural details while maintaining global contextual consistency, which is essential for accurate segmentation in H&E stained images. A further comparison with previously published WSI segmentation methods on the HuBMAP-2 dataset is provided in Table 11. This table includes standard UNet and Ensemble UNet models as reported in prior studies. GlomNet achieves the highest F1 score of 86.74%, outperforming Ensemble UNet (85.42%) and significantly surpassing the baseline UNet (77.19%). These results demonstrate that GlomNet not only improves upon conventional UNet-based approaches but also establishes superior performance compared to other recent segmentation frameworks designed for HuBMAP-2.

**Table 11:** Comparison of SOTA methods for WSI imagery on HuBMAP-2 dataset.

Model	Backbone	F1 (%)
Enet-b5 Unet (61)	EfficientNet-B5	77.19
Ensemble UNet (62)	EfficientNet-B7	85.42
GlomNet	LGC^2^-Former	**86.74**

Bold values indicate the highest achieved values. Underline values indicate the 2nd highest achieved values.

**Figure 11:**
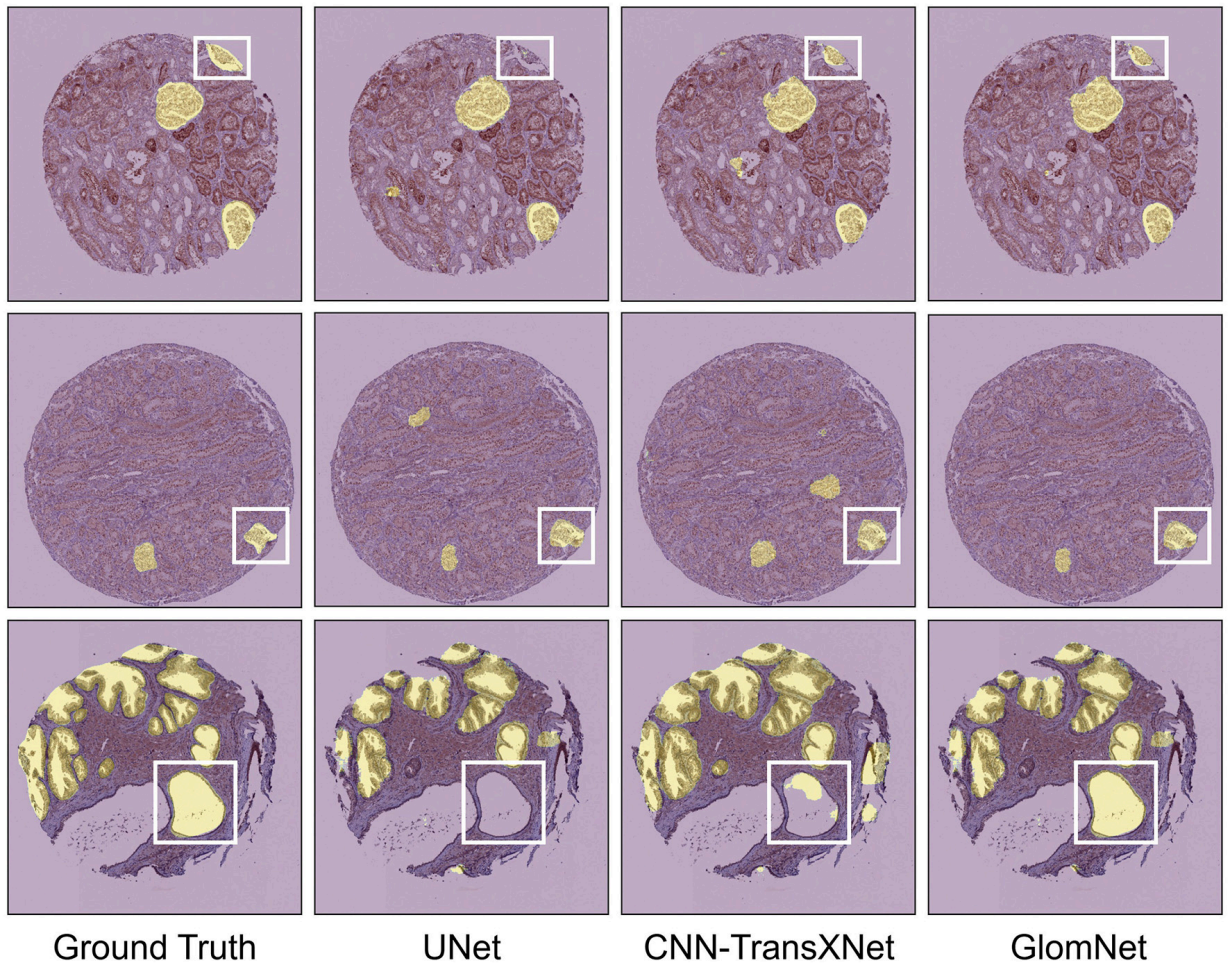
GlomNet qualitative comparison on HuBMAP-2.

Qualitative results further support the quantitative findings and are illustrated in Figure 11. For HuBMAP-2, UNet struggles with incomplete region coverage, inaccurate merging of adjacent structures, and poorly defined boundaries, leading to fragmented segmentation outputs. CNN-TransXNet improves boundary delineation and reduces some of these errors; however, it still fails to capture all regions of interest consistently. In contrast, GlomNet produces the most accurate and complete segmentation maps, with well-defined boundaries and minimal misclassification. These visual results clearly demonstrate the superiority of the proposed model in segmenting complex, multi-organ H&E stained WSIs, specifically identifying glomeruli, prostate glands, white pulp, crypts, and alveoli across various tissue samples. Overall, the experimental results on the HuBMAP-2 dataset confirm the effectiveness of GlomNet in challenging segmentation scenarios. The integration of the LGC^2^-Former backbone, HMFA-based skip connections, and the FRU decoder enables GlomNet to effectively capture both local structural details and broader contextual information. Consequently, GlomNet emerges as a robust and reliable solution for WSI segmentation tasks involving H&E stained medical images.

### 4.5 Discussion

The experimental results presented in this study demonstrate that GlomNet provides a robust and effective solution for glomeruli segmentation across diverse datasets and staining protocols. By evaluating the proposed model on HuBMAP-1, HuBMAP-2, and NEPTUNE datasets, which include PAS, H&E, SIL, and TRI staining techniques, the generalization capability of GlomNet has been thoroughly validated. These datasets introduce multiple challenges, such as high intra-class variability, complex tissue textures, and weak boundaries between glomeruli and surrounding regions. The consistent performance gains achieved by GlomNet across all these scenarios indicate its ability to effectively address the inherent complexities of histopathological image segmentation. One of the main strengths of GlomNet lies in its ability to jointly model local structural details and global contextual information. The LGC^2^-Former backbone plays a central role in this process by capturing fine-grained spatial features while simultaneously learning long-range dependencies within the tissue. This dual representation is particularly important for glomeruli segmentation, where precise boundary delineation must be preserved without losing the broader anatomical context. Such a balance is especially critical for H&E-stained images, where the visual contrast between glomeruli and background tissue is often subtle, making accurate segmentation challenging for conventional convolution-based models. The inclusion of HMFA-based skip connections further enhances the segmentation quality by enabling effective multi-scale feature fusion. By aggregating hierarchical features from different encoder levels, GlomNet ensures that important structural details are retained throughout the network. This design choice significantly improves boundary localization and reduces information loss that typically occurs in deep encoder-decoder architectures. In addition, the FRU decoder contributes to improved spatial reconstruction by employing an efficient upsampling strategy that mitigates common artifacts such as checkerboard patterns and blurred edges. As a result, the final segmentation maps produced by GlomNet exhibit sharper boundaries and more accurate object shapes compared to those generated by traditional upsampling techniques.

From a clinical perspective, the improvements offered by GlomNet have meaningful implications. Accurate glomeruli segmentation is a fundamental step in the diagnosis and assessment of renal diseases, including diabetic nephropathy, glomerulonephritis, and hypertension-induced kidney damage. Automated and reliable segmentation can support pathologists by reducing manual workload, improving reproducibility, and enabling quantitative analysis of glomerular morphology. The strong performance of GlomNet across different staining methods suggests that it can be effectively applied in real-world clinical environments, where staining protocols often vary depending on diagnostic requirements. Furthermore, the ability of GlomNet to generalize well across multiple datasets highlights its practical versatility. Unlike many existing approaches that require dataset-specific tuning or are limited to a single staining modality, GlomNet demonstrates stable performance across heterogeneous data distributions. This robustness makes it suitable not only for clinical deployment but also for large-scale research studies involving multi-center or multi-stain histopathological data. In summary, GlomNet represents a meaningful advancement in medical image segmentation by addressing critical challenges associated with glomeruli segmentation. Through the integration of the LGC^2^-Former backbone, HMFA skip connections, and the FRU decoder, GlomNet effectively captures both detailed structural information and global contextual cues. The resulting improvements in segmentation accuracy and robustness suggest that GlomNet has strong potential to support automated diagnostic workflows and contribute to improved clinical decision-making in nephrology.

## 5 Conclusion

Accurate segmentation of glomeruli in kidney histopathology images is essential for renal disease diagnosis but remains challenging due to limitations in current CNN-based methods. GlomNet effectively addresses these challenges through its innovative architecture. LGC^2^-Former tackles the issue of uniform processing that dilutes discriminative information and hinders effective separation from surrounding tissue by capturing both global context and fine-grained local details. It fuses these contexts, enhance spatial awareness and improving the representation of complex glomeruli structures. HMFA promotes richer feature extraction and effective multi-scale fusion, addressing the problem of incomplete feature fusion between layers. Additionally, FRU resolves artifacts commonly associated with traditional upsampling techniques, leading to clearer segmentation outcomes. Experimental validation shows that GlomNet achieves state-of-the-art performance across multiple benchmarks, including NEPTUNE, HuBMAP-1, and HuBMAP-2. Specifically, GlomNet attains F1 scores for glomeruli segmentation across various stains, including PAS (92.68%), H&E (83.83%), SIL (87.94%), and TRI (83.55%) on the NEPTUNE dataset. It achieves 96.09% for glomeruli segmentation on PAS stains in HuBMAP-1 and 86.74% for functional tissue unit (FTU) segmentation on HuBMAP-2, indicating its robustness in various histological conditions and significant potential for practical applications in kidney disease diagnosis and medical image analysis.

### 5.1 Future work

In future work, we plan to extend the model's capabilities to perform granular multi-class segmentation of specific glomerular pathologies, including healthy, segmentally sclerosed, and globally sclerosed units. This will involve expanding our dataset with expert-labeled pathological subtypes to further enhance the clinical utility of the model for chronic kidney disease assessment.

## Data Availability

Publicly available datasets were analyzed in this study. This data can be found here: https://www.kaggle.com/competitions/hubmap-organ-segmentation; https://www.kaggle.com/competitions/hubmap-kidney-segmentation.
